# Harnessing brain-derived extracellular vesicles to support RDoC-based drug development^☆^

**DOI:** 10.1016/j.nsa.2024.105406

**Published:** 2024-12-15

**Authors:** I. Magaraggia, J. Krauskopf, J.G. Ramaekers, Y. You, L. de Nijs, J.J. Briedé, R. Schreiber

**Affiliations:** a Department of Toxicogenomics, Faculty of Health, Medicine and Life Science, Maastricht University, P.O. 616, 6200, Maastricht, the Netherlands; b Department of Neuropsychology and Psychopharmacology, Faculty of Psychology and Neuroscience, Maastricht University, P.O. 616, 6200 MD, Maastricht, the Netherlands; c Department of Fundamental Neuroscience, Section Psychiatry & Neuropsychology, School for Mental Health and Neuroscience, Faculty of Health, Medicine and Life Science, Maastricht University, P.O. 616, 6200, Maastricht, the Netherlands; d Department of Neuroscience, Mayo Clinic, Jacksonville, 32224, FL, USA

## Abstract

The Research Domain Criteria (RDoC) framework offers a dimensional and transdiagnostic approach to understanding complex neuropsychiatric and neurological disorders, facilitating the development of novel therapeutics. However, the integration of cellular and molecular brain processes into the RDoC framework is hindered by the lack of adequate biomarkers. This review explores the potential of brain-derived extracellular vesicles (BDEVs) isolated from biofluids as a source of non-invasive mechanistic biomarkers in RDoC-based drug discovery. We provide an overview of BDEVs, including their classification, biological functions, and current methodologies for isolation and characterization. We then discuss studies that have investigated BDEV cargo as mechanistic biomarkers in CNS drug studies, focusing on target engagement, treatment response, and toxicity. Additionally, we address important considerations and open questions regarding the characterization and validation of BDEVs in neuroscience research. Special emphasis is placed on the use of miRNA cargo within BDEVs as molecular readouts, highlighting their stability, regulatory roles, and potential to reflect dynamic changes in brain function. Finally, the review proposes strategies to further investigate the use of BDEV miRNA cargo as molecular readouts, underscoring its ability to provide insights into the molecular mechanisms underlying drug effects and their relevance to CNS drug discovery within the RDoC framework. Despite existing methodological and conceptual challenges, BDEVs represent a promising tool for advancing RDoC-based drug discovery.

## Highlights

• Brain-derived extracellular vesicles (BDEVs) have potential as non-invasive molecular biomarkers for RDoC-based drug development.
• BDEV cargo analysis provides insights into molecular and cellular processes underlying target engagement, treatment response and toxicity.
• miRNAs inside BDEVs are valuable readouts for monitoring molecular response to drug treatment.
• Addressing standardization challenges is crucial for the broader application of BDEVs in RDoC-based drug development.

## Abbreviation list

ADEV: Astrocyte Derived Extracellular Vesicle
BBB: Blood Brain Barrier
BDEV: Brain Derived Extracellular Vesicle
CNS: Central Nervous system
CSF: Cerebrospinal Fluid
EV: Extracellular Vesicle
fMRI: Functional Magnetic Resonance Imaging
IHC: Immunohistochemistry
IA: Immunoaffinity
ISEV: International Society of Extracellular Vesicles
PET: Positron Emission Tomography
MDEV: Microglia Derived Extracellular Vesicle
miRNA: microRNA
MISEV: Minimal Information for Studies of Extracellular Vesicles
MoA: Mechanism of Action
MRS: Magnetic Resonance Spectroscopy
NDEV: Neuron Derived Extracellular Vesicle
PoC: Proof of Concept
PoM: Proof of Mechanism
RDoC: Research Domain Criteria
TEM: Transmission Electron Microscopy
UoA: Units of Analysis

## 1 Introduction to RDoC-based drug discovery and development

The central nervous system (CNS) poses significant challenges for drug discovery and development, partly due to limitations in current methods of disease classification for neuropsychiatric and neurological disorders (van der Doef et al., 2018). Traditional diagnostic frameworks such as the DSM-5 and ICD-11 categorize disorders based on clusters of symptoms and signs that cause clinically significant distress or impairment in functioning across social, occupational, and other critical areas (DSM-5, 2013; ICD-11, 2018). While clinically useful, these classification systems are limited in capturing the complex heterogeneity, comorbidity, and symptom overlap observed across different conditions (Borsboom et al., 2011; Tio et al., 2016). This lack of diagnostic precision often leads to treatments that do not fully address the array of symptoms within a given disorder and are frequently prescribed off-label for conditions with similar symptom profiles—e.g., the use of SSRIs across various mood disorders (Baldwin and Kosky, 2007; Jannini et al., 2022). To address these limitations, researchers are turning to integrative strategies that leverage multidisciplinary expertise and recent technological advancements.

**Fig. 1:**
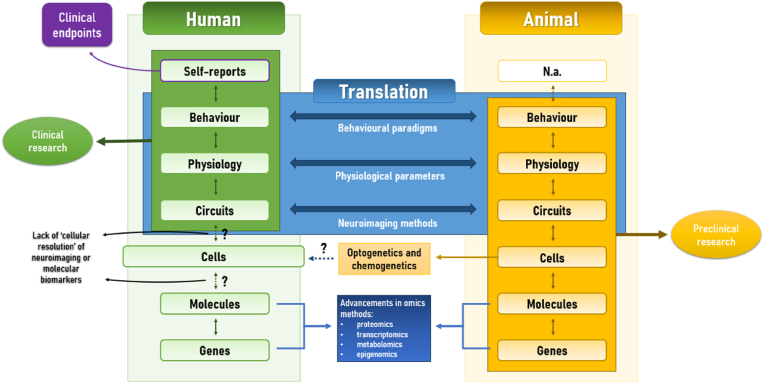
Translational and methodological considerations of RDoC-based CNS drug discovery. The Research Domain Criteria (RDoC) matrix can be used to characterize neurobehavioural domains of human functioning by integrating elements at various units of analysis, including self-reports, behaviour, physiology, circuits, cells, molecules, and genes.

One prominent approach is the National Institute of Mental Health's Research Domain Criteria (RDoC) initiative, which aims to deepen our understanding of brain disorders by focusing on neurobehavioral domains of basic human functioning and their biological underpinnings (Cuthbert, 2014; Insel et al., 2010). RDoC conceptualizes each neurobehavioral domain—or "construct"—through both psychological and neurobiological dimensions using a framework called the RDoC matrix. This matrix organizes various data levels, or "units of analysis" (UoA), including genes, molecules, cells, circuits, physiology, self-reports, and behaviors, in a way that captures their relationships over time and across contexts (Fig. 1). These UoA build on extensive data from fields such as cognitive neuroscience, neuropsychopharmacology, and basic neuroscience (Insel et al., 2010; Cuthbert, 2014; Morris et al., 2022).

By establishing relationships between each UoA within specific neurobehavioral domains, RDoC seeks to identify the neurobiological foundations of human behavior and to pinpoint distinct pathophysiological mechanisms underlying psychopathology, or “endophenotypes.” This approach thus offers a framework for translating preclinical findings into clinical practice and informing the development of targeted therapeutic strategies (Potter and Cuthbert, 2021; Tricklebank et al., 2021).

### 1.1 The role of biomarkers in RDoC-based drug discovery

Biomarkers—defined as measurable indicators of biological processes—play a critical role in contemporary drug discovery. They support essential steps in drug development, including optimizing dosages, monitoring drug efficacy and safety, and enabling patient stratification (Frank and Hargreaves, 2003; Kraus, 2018). In CNS research, biomarkers can offer insights into structural and functional changes in the brain under normal conditions (e.g., aging), pathological states (e.g., neurodegeneration), and in response to environmental exposures, such as drugs (Frank and Hargreaves, 2003; Kraus, 2018).

The Research Domain Criteria (RDoC) framework relies heavily on biomarkers to measure elements at each Unit of Analysis (UoA) in its matrix. Broadly, biomarkers are categorized by their methods in molecular, imaging, or physiological, or by their applications, such as diagnostic, prognostic, predictive, pharmacodynamic, safety, or surrogate biomarkers (Aronson and Ferner, 2017). Within RDoC-based drug discovery, using a diverse set of biomarkers allows researchers to demonstrate target engagement, pharmacodynamic response, and therapeutic efficacy across the various UoA. Importantly, the biomarker selection should align with a clear "line of sight" linking (1) identified clinical endophenotypes, (2) engagement with molecular targets, (3) associated biological circuits, and (4) biomarkers in early clinical development.

Over the past decades, technological advancements have made diverse biomarker methodologies available for measuring each Unit of Analysis (UoA) within the RDoC matrix in humans (Table 1). Foremost among these are neuroimaging methods, which allow for the objective, quantifiable, and longitudinal assessment of activity across and within brain regions in living humans (Phillips, 2012). Techniques such as positron emission tomography (PET), functional magnetic resonance imaging (fMRI), and magnetic resonance spectroscopy (MRS) have become integral to RDoC-based drug discovery and development (Howes and Mehta, 2021).

**Table 1:** Examples of methodologies currently employed in humans to measure elements at each *unit of analysis* of the RDoC *matrix*. At the *units of analysis* of *self-reports, behaviour, physiology, and circuits,* RDoC proposes a series of methodologies that can be employed for their measurements. However, RDoC does not provide any reference of the methods normally employed for the integration of elements at the *units of analysis* of *cells, molecules,* and *genes* (https://www.nimh.nih.gov/research/research-funded-by-nimh/rdoc/units). Therefore, it can only be assumed that the integration of these elements in humans mostly relies on histological and molecular analysis of biological samples, such as brain tissues biopsies and peripheral biofluids.

RDoC Units of Analysis	Current methodologies employed in humans	Examples
*Self-reports*	Patient-reported outcomes	questionnaires, interviews, rating scales
*Behavior*	Behavioral paradigms	Working memory tasks, decision-making tasks
*Physiology*	Vital parameters and circulating markers	Heart rate, pupil size, blood cortisol levels
*Circuits*	Neuroimaging	Functional magnetic resonance imaging, functional near-infrared spectroscopy
*Cells*	Microscopy analysis	Immunohistochemistry and transmission electron microscopy on postmortem brain tissue
*Molecules*	Expression levels of biomolecules (e.g., neurotransmitters, proteins)	Mass spectrometry or next-generation sequencing on biological samples
*Genes*	Genetics	Genome-wide association studies

Given that CNS disorders often involve disruptions in specific brain circuits, neuroimaging has been instrumental in identifying “circuitry biotypes”—patient subgroups with more homogeneous pathophysiology who may respond more favorably to targeted therapeutics. For instance, fMRI technology, combined with biotyping frameworks, has identified eight subtypes of depression, each reflecting unique dysfunctions in six major circuits (Hack and Williams, 2021). Furthermore, neuroimaging can yield essential insights into the pharmacodynamic properties of CNS drugs. This is demonstrated by various Proof-of-Mechanism (PoM) studies showing correlations between the extent of drug target occupancy or engagement, and corresponding changes in clinical outcomes (Kong et al., 2023; Sakayori et al., 2021; Egerton, 2021; Gilleen et al., 2021; Grimm et al., 2021; Kantrowitz et al., 2016). Notably, this approach has been successfully applied in recent RDoC-based clinical trials, such as the Fast-fail Trials in Psychosis Spectrum (FAST-PS; Kantrowitz et al., 2020) and the Fast-fail Trials in Mood and Anxiety Spectrum Disorders (FAST-MAS; Krystal et al., 2020; Box 1).

Box 1 **The FAST-MAS trial.**

The Fast-fail Trial in Mood and Anxiety Spectrum Disorders (FAST-MAS) applied the RDoC approach to evaluate a potential new treatment for anhedonia, a transdiagnostic core symptom of various mood disorder, depression and anxiety in particular (Krystal et al., 2020; Pizzagalli et al., 2020). Moreover, the trial is an example of applying the ‘fast-fail’ approach, which seeks to improve early-phase drug development methods by incorporating biomarker-based testing. The trial aimed to determine whether the novel kappa-opioid receptor (KOR) antagonist, JNJ-67953964, engages the intended target (proof-of-mechanism) and whether this engagement would lead to clinical improvement (proof-of-concept). Therefore, the trial used biomarkers of brain function, such as functional magnetic resonance imaging (fMRI), to measure the effects of the drug on the reward circuitry in patients and found that it increased ventral striatum activation during reward anticipation, compared to placebo. The results of the trial provided strong evidence to support further development of the drug for the treatment of anhedonia and highlighted the advantages of using RDoC-based approaches in CNS drug discovery and development. Meanwhile the drug advanced to Phase 3 clinical trials.

Alt-text: Box 1

Despite their considerable impact, neuroimaging methods alone do not fully address all dimensions of RDoC-based drug discovery. Conceptually, they have limited capacity to capture the cellular and molecular processes underlying brain function. Although PET and MRS can be used to assess specific neurobiological phenomena, such as microglial activation or shifts in neurotransmitter levels (Cai et al., 2019; Egerton, 2021; Pasanta et al., 2023; Tronel et al., 2017). Since drug discovery aims to pinpoint and address disrupted cellular and molecular pathways to restore CNS homeostasis, complementary methods are required to explore the underlying neurobiological mechanisms driving human behavior.

### 1.2 Molecular and cellular biomarkers for the CNS

At present, there are various method to measure molecular and cellular processes of the human brain. For example, morphological changes at the cellular level are typically investigated using techniques such as immunohistochemistry (IHC) and transmission electron microscopy (TEM) on brain tissue samples (Lewis et al., 2019; Sele et al., 2019; Waldvogel et al., 2006). Moreover, changes in protein expression, phosphorylation, and gene expression are usually measured by mass spectrometry (MS) or immunoassays, and sequencing techniques, respectively (Ferrer et al., 2008; Rajkowska and Stockmeier, 2013). These approaches have proven valuable in identifying ultrastructural synaptic changes and the distribution of toxic protein aggregates associated with neurodegenerative disorders, including Alzheimer's and Parkinson's disease (Arvanitakis et al., 2020; Griffiths et al., 1999; Nagatsu and Sawada, 2007; Reddy et al., 2010). Despite their contributions, these approaches are constrained by limited access to human brain tissue, which is typically available only postmortem or during surgery. This restricts their applicability to longitudinal studies and complicates attempts to correlate findings with other RDoC matrix elements, such as behavioral and physiological data.

In response to these limitations, researchers are increasingly exploring the use of peripheral biological sources to investigate CNS molecular and cellular changes more accessibly. Known as "liquid biopsy," this approach relies on the premise that qualitative or quantitative changes in the molecular composition of biofluids (e.g., blood, urine, saliva) can reflect (patho-)physiological changes in the brain (Poulet et al., 2019). These changes can be examined using both targeted and unbiased approaches. For instance, specific proteins can be measured using techniques such as Western Blot, ELISA, or ECLIA based on predefined hypotheses about the biological pathways of interest. However, this approach depends on prior knowledge of the pathways involved in the pathology under study. Consequently, there has been a shift toward unbiased omics approaches, including MS-based proteomics and next-generation sequencing (NGS) for RNA species, which have facilitated the identification of novel liquid biomarkers for diagnostic, prognostic, and treatment-response purposes (Abu-Asab et al., 2011; de Lange et al., 2017; Matthews et al., 2016).

Despite their potential, liquid biopsies present conceptual limitations in CNS research. First, establishing the CNS origin of biomolecules found in peripheral fluids is challenging. According to the Human Protein Atlas, only 10% of human genes are expressed exclusively in the CNS, which means that peripheral sources may contribute to most biomolecules detected in biofluids (Thul and Lindskog, 2018). Second, the blood-brain barrier (BBB) restricts the transfer of biomolecules from the CNS to peripheral circulation, potentially leading to peripheral levels that do not accurately reflect CNS concentrations (Koníčková et al., 2022). To address these limitations, researchers often turn to cerebrospinal fluid (CSF), which more directly reflects CNS molecular changes (Milà-Alomà et al., 2019; Slavik and Dolezal, 2012). However, CSF collection is invasive and associated with risks such as nerve injury and post-lumbar puncture headache, requiring trained personnel and medical supervision (Niemantsverdriet et al., 2015). Additionally, some studies have found that CSF biomarkers do not always outperform blood-based biomarkers in detecting disease states (Forgrave et al., 2019). Therefore, there remains a critical need for improved, non-invasive methods to enhance the sensitivity and specificity of molecular liquid biomarkers for tracking cellular and molecular CNS processes.

### 1.3 The need for non-invasive biomarkers in RDoC-based drug discovery

The absence of accessible and sensitive biomarkers has led most RDoC-based human studies to overlook cellular and molecular aspects of brain function. For instance, the MIND-SET study (Measuring Integrated Novel Dimensions in Neurodevelopmental and Stress-Related Mental Disorders) is a large planned RDoC study designed to identify shared endophenotypes between neurodevelopmental and stress-related disorders within a large patient cohort (van Eijndhoven et al., 2022). While this study includes data across multiple levels—including genetics, physiology, neuropsychology, system-level neuroimaging, behavior, self-reports, and neurocognitive paradigms—it lacks measures of molecular and cellular processes. Similarly, Fornster et al. (2022) conducted a study examining shared behavioral and cognitive abnormalities across various RDoC domains in a heterogeneous psychiatric population using validated behavioral test batteries, yet did not incorporate biomarkers for cellular or molecular events. RDoC drug studies, such as FAST-MAS and FAST-PS, have also focused exclusively on neuroimaging biomarkers to demonstrate target engagement without molecular or cellular readouts. While the depth and scope of this type of pioneering studies represent invaluable contributions to advancing our understanding of mental disorders, the inclusion of molecular readouts could further enhance their impact by helping to establish clear links between each Unit of Analysis of the RDoC matrix.

For this purpose, the RDoC framework still relies heavily on cross-species, or “vertical translation,” neuroscience approaches (Fig. 1). Preclinical models remain the primary tools for closely monitoring molecular processes in the living brain, utilizing methods such as chemogenetics and optogenetics (Deisseroth, 2015; Roth, 2016), or conditional gene knockouts (Nelson, 2015). Crucially, a defining aspect of translational neuroscience approaches is the use of behavioral paradigms with high translational validity, which can be applied across species and strongly correlate with self-reported measures in humans. However, translating findings from animals to humans remains challenging which limits the ability to establish clear links between molecular aspects of brain functioning and human behavior according to the RDoC approach (Silverman et al., 2020; Tricklebank et al., 2021).

As such, it is clear that the RDoC framework may greatly benefit from the development novel molecular biomarker strategies in humans without relying on postmortem brain tissues analyses or translational approaches. One prominent solution would be to increase the sensitivity and specificity of liquid biopsies for the brain. Developing a method that allows to track down the cellular origins of circulating biomarkers would allow to reduce the signal ‘noise’ resulting from peripheral sources. Analogous to the “spatial resolution” of neuroimaging markers, this concept may be referred to as “cellular resolution” of circulating brain biomarkers. As a result of increased specificity and sensitivity, such biomarkers may provide mechanistic insights into cellular and molecular processes underlying both normal and pathological human behavior, thus greatly advancing RDoC-based drug discovery and development.

For this purpose, extracellular vesicles (EVs) found in biofluids have emerged as promising new biomarker strategy. EVs are nano-sized vesicles of varying sizes, cargo, and cell-specific surface markers, secreted into the extracellular environment through different cellular mechanisms. These vesicles carry a range of cytoplasmic and cell membrane components selectively loaded within them that seem to mirror the physiological state of the parental cell. Therefore, scientist have explored to possibility to use the cargo of peripheral brain-derived EVs (BDEVs) to measure molecular aspects of brain functioning in humans. In the following section, we introduce EVs, their classification, and general functions within the human body, followed by a discussion of their potential as mechanistic biomarkers in RDoC-based drug discovery (Fig. 1).

Note: As with many rapidly expanding research fields, EV studies face challenges related to the lack of standardization in defining, classifying, and reporting experimental findings. To address this, the International Society for Extracellular Vesicles (ISEV) was established to support research advancement, knowledge dissemination, and collaboration within the EV field (Araldi et al., 2012). Notably, ISEV has provided guidelines for EV definition and classification, as outlined in the "Minimal Information for Studies of Extracellular Vesicles (2023)" (MISEV2023), that builds upon previous versions of the document from 2014 to 2018 (Welsh et al., 2024; Théry et al., 2018). This review adheres to the latest MISEV2023 guidelines for EV nomenclature and classification.

## 2 Introduction to extracellular vesicles in biomedical research

### 2.1 Definition, classification, and general function

**Fig. 2:**
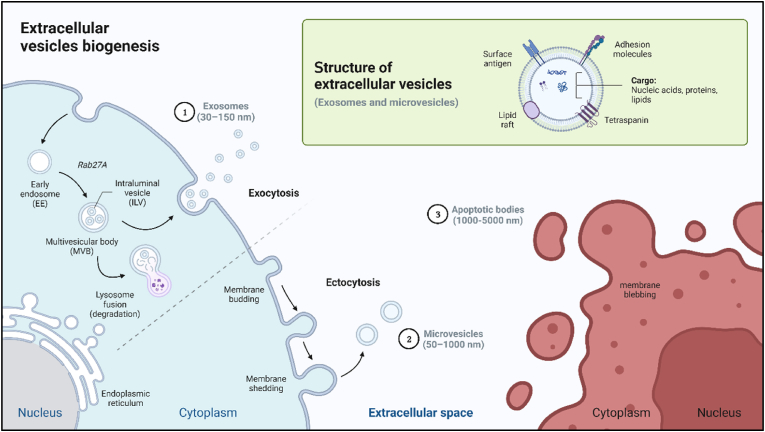
Extracellular vesicles (EVs) biogenesis and structure. In general, EVs are particles delimited by a double lipid membrane that express a vast array of biomolecular cargo, including nucleic acids, proteins, and lipids both inside and on their surface membrane. The major EV subtype are exosomes (1), whose biogenesis is complex and involves multiple steps. First, inward budding of the endosomal membrane results in the formation of multivesicular bodies (MVBs) containing intraluminal vesicles (ILVs), i.e. the precursor of exosomes (Akers et al., 2013; Hessvik and Llorente, 2018). Subsequently, the fusion of the outer MVB membrane to the plasma membrane leads to the release of exosomes into the extracellular space by exocytosis. This process is mediated by either the endosomal sorting complex required for transport (ESCRT), or an orchestrated action of cholesterol, ceramide, and lipid rafts. Microvesicles (2), also referred to as microparticles or ectosomes, are the second major type of EVs, that result from the outward budding of the plasma membrane, i.e. ectocytosis (Sedgwick & D'Souza-Schorey, 2018). In terms of size, microvesicles are generally larger than exosomes and represent a more heterogeneous population, including ectosomes, oncosomes, and microparticles. In fact, microvesicles possess sizes ranging from 50 to 1000 nm, whereas exosome possess sizes between 50 and 150 nm (van Niel et al., 2018). Finally, apoptotic bodies (3) are the third subtype of EVs that are characterized by a distinct biogenetic pathway involving the disintegration of the plasma membrane of cells. Compared to exosomes and microvesicles, apoptotic bodies are significantly larger particles, with sizes sometimes reaching several microns. Created with BioRender.com.

Extracellular vesicles (EVs) are broadly defined as particles naturally released from cells, encapsulated by a lipid bilayer, and unable to replicate due to their lack of a functional nucleus (Théry et al., 2018). In fact, EVs are a diverse group of membrane-bound particles secreted by all cell types in the human body (Abels and Breakefield, 2016). The classification of EVs has been subject to ongoing debate, with multiple classification frameworks proposed (Y. Li et al., 2023; Margolis and Sadovsky, 2019). Nonetheless, the MISEV guidelines endorse a widely accepted classification that divides EVs into three main subtypes: exosomes, microvesicles, and apoptotic bodies, distinguished primarily by their biogenesis and physicochemical characteristics (Fig. 2). While these subtypes have unique biogenetic and physical features, it is important to note that their properties can vary depending on the originating cell type and environmental conditions (van Niel et al., 2018). Consequently, careful consideration of EV subtypes is essential in the context of their cellular source and isolation methods (see next section).

In the last decades, research has demonstrated that extracellular vesicles (EVs) cover a plethora of biological functions within the body. Although they were thought initially to function solely as a cellular disposal mechanism to remove unwanted materials (Pan and Johnstone, 1983), subsequent studies revealed a more prominent role for EVs as mediators of intercellular communication (Frühbeis et al., 2012, 2013; Luga et al., 2012; Raposo and Stahl, 2019). EVs contain a diverse range of biomolecules, including lipids, sugars, proteins, and various forms of genetic material, such as DNA and both coding and non-coding RNA species (Fig. 2). Through the transport and delivery of these molecular cargos, EVs released from a donor (or parental) cell can modulate the phenotype of recipient cells upon uptake (Latifkar et al., 2019). Recipient cells can take up EVs through multiple pathways, including endocytosis, phagocytosis, membrane fusion, and less frequently through receptor-mediated uptake, lipid raft-mediated uptake, micropinocytosis, and tunneling nanotubes (Mulcahy et al., 2014). This capability of EVs to influence recipient cell phenotypes supports a wide range of physiological and pathological processes involving intercellular communication. These processes include inflammation, immune responses, cell repair, and stages of cell growth such as proliferation, differentiation, and maturation (Raposo and Stahl, 2019). Recent state-of-the-art reviews have detailed EV involvement in various pathologies, including renal, cardiovascular, and autoimmune disorders (Pomatto et al., 2017; Saheera et al., 2021; Shao et al., 2020; Tian et al., 2020).

EVs also play a crucial role in the central nervous system (CNS). Brain-derived EVs (BDEVs) facilitate communication between different CNS cell types, including neurons, microglia, and astrocytes, and contribute to a range of both physiological and pathological brain functions. For example, BDEVs are integral to adult neurogenesis and neuroplasticity, as they deliver biomolecules such as neurotrophins and specific microRNAs (miRNAs) throughout brain systems (Antoniou et al., 2023; Zhang and Xu, 2022). Furthermore, EVs derived from stem cells promote the maintenance, proliferation, and neuronal differentiation of neural stem and progenitor cells in the dentate gyrus of the hippocampus (Losurdo and Grilli, 2020). Additionally, miRNA inside BDEVs appeared to be implicated in the effects of brain-derived neurotrophic factor (BDNF) on both the morphological and functional aspects of neuroplasticity (Antoniou et al., 2023). In line with these observations, alterations in BDEV signaling have been associated with neuropsychiatric disorders marked by impaired neuroplasticity, such as depression and schizophrenia (Y. Li et al., 2023). Moreover, BDEVs seem to be involved in other pathological brain processes, such as protein aggregation and misfolding, neuronal damage, abnormal synaptic communication, and blood-brain barrier dysfunctions (Basso and Bonetto, 2016; Pegtel et al., 2014; You and Ikezu, 2019). Consequently, BDEVs have been linked to various neurodegenerative diseases, including Parkinson's and Alzheimer's disease (Hill, 2019; Quek and Hill, 2017; Muraoka et al., 2020). Taken together, these findings suggest a prominent role for BDEVs in both healthy and disease CNS function.

### 2.2 Extracellular vesicles isolation and characterization methods

#### 2.2.1 Extracellular vesicles isolation

Isolating extracellular vesicles (EVs) from biological fluids and tissues is a crucial step for investigating their composition, functions, and potential diagnostic or therapeutic applications. Various methods have been developed and optimized for EV isolation, each leveraging specific physical and chemical properties of EVs and therefore offering distinct advantages and limitations. Common techniques include serial centrifugation (SC), ultracentrifugation (UF), size exclusion chromatography (SEC), precipitation methods, immunoaffinity-based (IA) methods, and microfluidic technologies. The principles, strengths, and limitations of these methods have been comprehensively reviewed (Bağcı et al., 2022; Coumans et al., 2017; Lai et al., 2022; Martins et al., 2023). Selecting an appropriate EV isolation method depends on the research objective, sample type, and intended downstream applications (Zhang et al., 2023).

A significant advancement in EV isolation is the identification of surface proteins specific to EV subtypes or particular cellular origins. For instance, exosomes are highly enriched with the tetraspanins CD9, CD63, and CD81 (Andreu and Yáñez-Mó, 2014), while EVs from endothelial cells or platelets exhibit high levels of CD105 and CD41, respectively (Wang et al., 2016). Techniques such as proteomic analysis, flow cytometry, super-resolution imaging, and functional assays have been used to identify these markers (Fauré et al., 2006; Yang et al., 2017; You et al., 2023). These methods have also facilitated the identification of specific markers for brain-derived EVs (BDEVs), with several markers found to be highly enriched in EVs originating from CNS cell types, including neurons, microglia, astrocytes, and oligodendrocytes (Table 2).

**Table 2:** List of studies that identified surface protein markers for the isolation of brain-derived extracellular vesicles from neurons, microglia, astrocytes, and oligodendrocytes. ACSA-1: astrocyte cell surface antigen-1; CD11b: integrin alpha M subunit; CNPase = 2,3-cyclic nucleotide-3-phosphodiesterase; GAP-43: Growth Associated Protein-43; GFAP = glial fibrillary acidic protein; GLAST = glutamine aspartate transporter; GluR2/3: Glutamate Receptor 2/3; L1CAM = L1 cell adhesion molecule; MOG = myelin oligodendrocyte glycoprotein; NCAM = neuronal cell adhesion molecule; NMDAR2A = N-methyl-D-aspartate receptor subunit 2A.

CNS cell type	Identified surface markers	References
*Neuron*	ATP1A3	You et al. (2023)
	L1CAM (CD171)	(Mustapic et al., 2017; Shi et al., 2014)
	NCAM	Fiandaca et al. (2015)
	NMDAR2A	Tian et al. (2022)
	GluR2/3	Yousif et al. (2022)
	GAP43	Volpert et al. (2022)
*Microglia*	LCP1	You et al. (2022)
	CD11b	Cohn et al. (2021)
	TMEM119	Kumar et al. (2021)
*Astrocytes*	GLAST (EAAT1)	Goetzl et al. (2016)
	ACSA-1	Burrows et al. (2023)
	GFAP	Willis et al. (2017)
	Aquaporin 4	Wallensten et al. (2021)
	ITGA6	You et al. (2022)
	LRP1	You et al. (2022)
*Oligodendrocytes*	CNPase	Yu et al. (2020)
	MOG	Dutta et al. (2021)
	LAMP-2	You et al. (2022)
	FTH1	You et al. (2022)

Building on this discovery, researchers have developed immunoaffinity (IA) methods that selectively isolate specific EV subpopulations based on surface markers. These methods include immunoprecipitation and immunocapture techniques that use biotinylated antibodies capable of binding covalently to solid surfaces (e.g., plates or chips) or semi-fluid devices (e.g., magnetic beads) coated with streptavidin (Kang et al., 2017; Liangsupree et al., 2021). Although IA techniques are typically low-throughput, time-intensive, and require costly equipment, they offer a major advantage by yielding high-purity EV samples compared to conventional isolation methods such as ultracentrifugation (UF), size exclusion chromatography (SEC), or precipitation. An optimal strategy involves a two-step process: first, total EVs are isolated using precipitation or SEC, followed by IA-based enrichment of specific EV subtypes. This approach has become increasingly popular for isolating and characterizing brain-derived EVs (BDEVs) from human biofluids (Cano et al., 2023; Dutta et al., 2023).

In addition to their practical limitations however, IA methods face important conceptual and methodological limitations. A primary concern is the specificity of selected markers, as non-target cellular sources may also express these markers, complicating the isolation of pure EV subtypes. Additionally, antibody cross-reactivity and the presence of soluble protein forms within the sample can interfere with the IA process, potentially compromising the purity of the isolated EV population. Large sample volumes may also be necessary to isolate EV subpopulations via IA methods (Ma et al., 2019). In the case of BDEVs, the L1 adhesion molecule (L1CAM) exemplifies these issues (Box 2). Despite these limitations, IA methods have been extensively employed to isolate BDEVs for research purposes. (see Box 2).

Box 2 **L1CAM as surface marker for neuronal EV.**

L1 cell adhesion molecule (L1CAM) is a glycoprotein involved in cell-cell adhesion, neuronal migration, and axonal growth, playing a key role in nervous system development and function. Although L1CAM is widely used as a marker for neuron-derived EVs (NDEVs) due to its predominant expression in neurons (Gomes and Witwer, 2022), it is also expressed in non-neuronal cells and in organs outside the CNS (Angiolini et al., 2019; Hlavin and Lemmon, 1991), which can confound NDEV studies relying on this marker. Moreover, L1CAM is a glycoprotein that undergoes various post-translational modifications (Haspel and Grumet, 2003; Samatov et al., 2016), and it is unclear which specific variants are targeted by commercially available anti-L1CAM antibodies. These observations underscore the need to include rigorous control conditions when characterizing cell-specific EVs to prevent antibody cross-reactivity and to remove soluble protein forms prior to IA to reduce potential interference.

Alt-text: Box 2

#### 2.2.2 Extracellular vesicles characterization

Given the heterogeneity of EVs and the complexity of their sources, characterizing EV samples to assess purity is a crucial step in EV research. According to MISEV guidelines, EV characterization should employ orthogonal methods to evaluate: 1) particle concentration and size, 2) EV morphology, 3) total protein, lipid, and RNA concentrations, and 4) EV protein composition.

Measuring particle size and morphology is one of the most essential steps in EV characterization, as it provides valuable information regarding sample purity, despite some overlap in EV subtype sizes (Fig. 2). Size is commonly measured using nanoparticle tracking analysis (NTA), flow cytometry (FC), or dynamic light scattering (DLS), which can also quantify particle concentration (Szatanek et al., 2017; Xu et al., 2016). Assessing particle morphology is equally important to accurately detect EVs in the sample and exclude contaminants like lipoproteins and cell debris. Techniques such as transmission electron microscopy (TEM), scanning electron microscopy (SEM), and atomic force microscopy (AFM) are typically used for this purpose (Szatanek et al., 2017).

Another critical step in EV characterization involves analyzing their molecular content. Although no universally accepted markers for EV subtypes exist, certain protein markers can provide insights into the type and origin of EVs. For instance, the presence of proteins CD9, CD63, CD81, and HSP70 serves as a marker for exosomes (Bağcı et al., 2022; Lai et al., 2022). These protein markers are typically measured through biomolecular techniques such as Western blotting or ELISA (Tiwari et al., 2021). Additionally, detecting cell-specific markers in EVs is essential; for example, neuronal EVs can be identified by the presence of neuron-specific enolase, synaptophysin, neurofilament proteins, and microtubule-associated proteins (Dutta et al., 2023; Mustapic et al., 2017). Alternatively, cell-specific RNA species can be detected using quantitative polymerase chain reaction (qPCR), microarrays, or next-generation sequencing (NGS); for example, miR-9 and miR-124 are recognized as neuronal miRNA markers (Saeedi et al., 2021). Measuring protein and RNA content also helps to control for contaminants such as extracellular matrix components or EVs from unrelated cell types. For example, when isolating EVs from plasma or serum, the absence of blood contaminants like albumin and immunoglobulin G should be verified (Dong et al., 2020; Holcar et al., 2021), whereas the presence of collagens and fibronectin is typically checked in EVs isolated from cell culture medium (Benedikter et al., 2017; Dong et al., 2020).

Although current methods provide valuable insights into the types of EVs present within samples, challenges remain due to overlapping EV sizes and the lack of specific biogenesis markers for exosomes or microvesicles. Consequently, the MISEV guidelines recommend against using specific EV subtype names in publications (Théry et al., 2018). Instead, they suggest employing “operational terms” that reflect the context of EV isolation and characterization; for instance, EVs isolated via immunoaffinity methods could be classified based on the specific surface marker used (e.g., L1CAM+). To further support transparency and standardization in EV research, scientists are encouraged to document their experimental methods and protocols on databases like EV-TRACK (https://evtrack.org/), which enables comprehensive tracking of extracellular vesicle research (Van Deun et al., 2017).

### 2.3 The application of extracellular vesicle cargo in biomedical research

In recent years, researchers have explored various applications of extracellular vesicles (EVs) in biomedical research. Among these applications, the use of EVs as “liquid biopsies” for investigating molecular mechanisms underlying both pathological and physiological processes has received particular attention. Since EVs are released by all cell types within the body and circulate in peripheral fluids such as blood, saliva, urine, and tears (Raposo and Stahl, 2019), they are accessible through minimally or non-invasive sampling methods, making them ideal for longitudinal studies. Moreover, when validated cell-specific markers are selected, the origin of EVs isolated through immunoaffinity methods is often known, thus allowing researchers to draw conclusions about the molecular status of their tissue of origin based on EV cargo.

The use of EVs as liquid biopsies rests on the fundamental assumption that EV cargo reflects the (patho-)physiological state of the parental cell. In fact, various preclinical studies have demonstrated significant correlations and overlap between the molecular content of EVs and that of their tissue or cell population of origin. For example, ovarian cancer cells and glioblastoma cells produce EVs with tumor-related proteins and RNA expression patterns similar to those in the originating cells (Im et al., 2014; Runz et al., 2007; Skog et al., 2008). Additionally, proteomic content in urinary EVs from rats and patients with Gitelman syndrome correlated strongly with kidney tissue profiles, particularly for membrane proteins, highlighting EVs’ potential to mirror specific cellular states (Wu et al., 2021; Sung et al., 2021). Although the extent to which EV cargo accurately reflects the molecular state of parental cells remains to be fully clarified, especially for the CNS, these findings support the use of EVs as liquid biopsies.

Researchers have investigated the potential of using the cargo of brain-derived extracellular vesicles (BDEVs) in blood as “liquid biopsies” to explore the neurobiological basis of neurological and neuropsychiatric disorders. BDEVs can cross the blood-brain barrier (BBB) and enter systemic circulation; furthermore, the identification of brain-specific markers has enabled their isolation via immunoaffinity (IA) methods (Dutta et al., 2023). This approach offers a non-invasive means of examining molecular and cellular mechanisms underlying brain function in both healthy individuals and patients with CNS disorders. BDEVs have demonstrated a wide range of applications, serving as diagnostic, prognostic, and disease progression biomarkers for neurodegenerative and neuropsychiatric diseases.

While these applications have been reviewed extensively elsewhere (Badhwar and Haqqani, 2020; Cano et al., 2023; Dutta et al., 2023; Guedes et al., 2020; Oraki Kohshour et al., 2023), this review will focus specifically on the use of BDEV cargo as a source of mechanistic biomarkers for assessing the effects of pharmacological interventions in the CNS.

## 3 Drug studies investigating the use of brain-derived extracellular vesicles cargo as mechanistic biomarkers for CNS drug discovery

The cargo of extracellular vesicles (EVs) offers a valuable source of mechanistic biomarkers for CNS drug discovery, as it may reflect pharmacologically induced changes in molecular and cellular processes related to both the therapeutic and toxic effects of treatments (Holman et al., 2016; Motawi et al., 2018; Pan et al., 2021). This approach provides researchers with a novel tool to investigate the pharmacodynamic properties of drugs in the brain at a molecular level, addressing the limitations of neuroimaging techniques. To our knowledge, only relatively few studies have examined changes in the molecular cargo of brain-derived EVs (BDEVs) as mechanistic biomarkers in both human and animal drug studies (Table 3). A detailed description of the study protocols and findings can be found in the appendix. Based on findings from these studies, we outline a series of key methodological and conceptual considerations below.

**Table 3:** Overview of animal and human drug studies employing the molecular cargo of brain-derived extracellular vesicles (BDEVs) as mechanistic biomarker in CNS drug discovery and development. Applications of BDEV cargo as mechanistic biomarker include measures of treatment response, neurotoxicity, and pharmacodynamic responses such as signal transduction and drug cell effect. Abbreviations: L1CAM = L1 cell adhesion molecule; ACSA-1 = astrocyte cell surface antigen; NTA = nanoparticle tracking analysis; WB = Western Blot; FC = flow cytometry; NDEV = neuron-derived extracellular vesicle; GLAST = Glutamate Aspartate Transporter 1; ADEV = astrocyte-derived extracellular vesicles; NGS = next-generation sequencing; fMRI = functional magnetic resonance imaging; MDD = major depressive disorder; ADT = antidepressant treatment; SEC = size-exclusion chromatography; TEM = transmission electron microscopy; CD9 = tetraspanin 9; CD81 = tetraspanin 81; HSP70 = heat shock protein 70; SNAP25 = Synaptosome Associated Protein 25; BIP = Binding immunoglobulin protein; qPCR = quantitative polymerase chain reaction; PD = Parkinson's disease; RCT = randomized clinical trial; ECLIA = Enhanced “Chemiluminiscence” Immunoassay; MoA = mechanism of action; TMEM119 = transmembrane protein 119; ELISA = Enzyme-linked immunosorbent assay; RT-PCR = real-time polymerase chain reaction; LC-MS = liquid chromatography coupled to mass spectrometry; DC = differential centrifugation; TSG101 = Tumor susceptibility gene 101; LRKK2 = Leucine-rich repeat kinase 2. See supplement for a full description of these studies.

Study	Subjects	Design	Treatment	Time of sample collection	Sample type	Total EV isolation	EV subpopulation	Characterization methods	BDEV cargo readout	Validation	Main findings
Burrows et al. (2022) & 2023	20 healthy human subjects	Randomized cross-over design	Placebo, 200 mg, and 600 mg Ibuprofen	5-h post-administration	Serum	ExoQuick	L1CAM+, ACSA-1+	NTA, FC, WB (L1CAM for NDEVs, GLAST for ADEVs)	miRNA expression (NGS)	N.a.	Dose-dependent increase in the expression levels of *a priori* selected miRNAs involved in neuro-inflammatory pathway in both NDEVs and ADEVs, which are associated with changes in reward-processing and brain activity (fMRI)
Saeedi et al. (2021)	40 MDD patients (20 responders and 20 non-responders to ADT) and 20 healthy controls	Between-subject design (n = 20)	8-weeks of recommended doses of the SSRI Escitalopram	End of treatment period	Plasma (type not specified)	qEV SEC columns (Izon Science)	L1CAM+	TEM, WB (CD9, CD81, HSP70 for exosomes, L1CAM, SNAP25, BIP for NDEVs), NGS (miRNA binding motifs, miR-9 expression, and overlap between NDEV and brain tissue miRNAs)	miRNA expression (NGS)	qPCR, independent cohort samples, human postmortem brain tissue, in vitro models	ADT responses is associated with larger NDEV sizes, and was predicted by the combination of several miRNAs which are associated with brain-enriched molecular pathways
Athauda et al. (2019)	60 PD patients (age 25–75)	Placebo-controlled RCT; between-subject design (n = 30)	Placebo or 2 mg Exanatide once weekly for 48 weeks	Baseline, 12- and 48-weeks post-administration, 12-weeks after drug withdrawal	Unspecified	ExoQuick	L1CAM+	Unspecified	Protein expression (ECLIA)	N.a.	Treatment and time-dependent changes in expression levels of phosphorylated and non-phosphorylated protein related to drug MoA that correlated with clinical outcome
Kumar et al. (2021)	6 adult male cynomolgus monkeys	Between-subject design with control group (n = 3)	Long-term oxycodone self-administration	3 years after chronic self-administration	K3-EDTA plasma	ExoQuick	L1CAM+, GLAST+, TMEM119+	NTA, WB (CD9, CD31, HSP70, Calnexin), FC	Protein expression (ELISA), miRNA expression (RT-PCR), Proteomics (LC-MS)	In vitro validation of proteomic results on associated biological pathways	Changes in protein and miRNA expression levels that correlate with gray matter loss and are linked to neuroinflammatory pathways
Taymans et al., 2023	3 adult female cynomolgus monkeys	Within-subject	5 mg/kg of the LRRK-2 kinase inhibitor PF-360	2- and 6-h post-administration	Urine (using catheter)	DC	N.a. (urine EVs)	NTA, TEM, WB (TSG101)	Phosphorylation of Rab and LRRK2 (sensitive WB)	LRKK2	Reduced Rab phosphorylation rates at 2-h post-administration, which were not maintained at the 6 h timepoint. Phosphorylated LRKK2 levels were out of the limit of detection.

### 3.1 Methodological considerations of using BDEVs

As previously mentioned, the methods of EV isolation and characterization strongly influence the interpretation of the findings. In this regard, important differences are present between studies which must be considered before comparing results. These include differences in the selected BDEV subtype, methods of isolation, and molecular readout.

#### 3.1.1 BDEV type and origin

All studies mainly focused on the molecular mechanisms underlying the effects of pharmacological interventions on a specific subtype of brain-derived EVs (BDEVs), namely neuron-derived EVs (NDEVs), using L1CAM as a surface marker. This aligns with the common assumption that therapeutic mechanisms of most CNS drugs are mediated by neurons and their receptor pools. Only two studies expanded their analysis to include additional BDEV subtypes, such as astrocyte-derived EVs (ADEVs) identified by ACSA1 or GLAST markers, and microglia-derived EVs (MDEVs) marked by TMEM119 (Burrows et al., 2023; Kumar et al., 2021).

While L1CAM's specificity as a marker for NDEVs has been well validated, the same cannot yet be said for markers of ADEVs and MDEVs (Dutta et al., 2023). For instance, according to data from the Human Protein Atlas, TMEM119 is also expressed in the liver, respiratory system, lymphoid tissue, and gastrointestinal tract, in addition to microglia (Thul and Lindskog, 2018). Future studies should therefore address the specificity of markers used for BDEV isolation to reinforce their application as mechanistic biomarkers in CNS drug discovery. Rather than focusing on a single BDEV subtype, examining panels of multiple subtypes in response to pharmacological interventions may yield new insights into drug safety and efficacy mechanisms.

When studying peripheral BDEVs as CNS biomarkers, a key assumption is their CNS origin. However, universally accepted methods to validate this origin are currently lacking, and researchers are thus encouraged to develop their own approaches. While most studies omit these validation steps, Saeedi et al. (2021) employed innovative strategies to confirm the neuronal origin of plasma-derived NDEVs, including the use of postmortem brain tissue, miRNA brain motif enrichment, and EVs isolated from saliva. Future studies should consider employing similar methods when feasible, while continued efforts to develop new validation techniques are strongly encouraged.

#### 3.1.2 BDEV cargo and analysis

Most studies used similar methods for BDEV isolation, with one notable exception. While the majority followed the protocol by Mustapic et al. (2017) for isolating NDEVs, which involves an initial isolation of total EVs using the ExoQuick precipitation kit (Systems Bioscience), the escitalopram study employed qEV size exclusion chromatography columns (Izon Science) to isolate total EVs (Saeedi et al., 2021). Although all studies used the same anti-L1CAM antibodies (clone 5G3, eBioscience) and immunoaffinity (IA) procedures to enrich for L1CAM-associated EVs, differences in total EV isolation methods may account for the variations observed in average NDEV sizes across studies.

Variations in total EV isolation techniques may influence the efficiency and purity of subsequent IA methods. For instance, different isolation methods can vary in their tendency to co-precipitate soluble L1CAM or in their impact on the surface properties of EVs, potentially affecting antibody binding to L1CAM-associated EVs (Gomes and Witwer, 2022). However, most studies lack a preliminary characterization of total EV size and concentration prior to enrichment, making it difficult to draw firm conclusions. This brings to light another key methodological difference: the characterization of BDEV samples. Although nearly all studies assessed exosomal markers via Western blot or ELISA, the specific markers chosen varied. Additionally, positive and negative markers for exosomes and BDEVs were not consistently evaluated in both the initial EV populations and EV-depleted fluids. Overall, these observations underscore the need to standardize BDEV isolation and characterization protocols to facilitate better comparability between studies.

Studies vary in the molecular cargo selected as biomarker readouts, as well as in their measurement, analysis, and validation methods. Some studies focus on preselected panels of proteins or miRNAs (biased approach), while others examine entire proteomes or transcriptomes using mass spectrometry (MS) or next-generation sequencing (NGS) (unbiased approach). In the latter case, bioinformatic analysis methods also differ, such as the choice of target prediction algorithms for miRNA data interpretation.

Validation methods also vary widely across studies. Some studies lack validation steps, including the use of orthogonal methods to confirm the same readouts with different techniques (e.g., using qPCR to validate NGS findings). In contrast, Saeedi et al. (2021) strengthened their findings on altered physicochemical properties of NDEVs post-drug treatment by using multiple validation approaches, including orthogonal techniques, independent patient cohorts, and in vitro models.

### 3.2 Conceptual considerations to the use of BDEV cargo as mechanistic biomarkers

Despite the limited number of studies and methodological differences that hinder direct comparisons, valuable conceptual insights can be drawn based on these studies regarding the use of BDEV cargo as a mechanistic biomarker in CNS drug discovery.

First, these studies indicate that drug exposure may be associated with changes in the physicochemical properties of peripheral BDEVs. Findings consistently show that both single and chronic treatments lead to acute and long-term alterations in the expression levels of proteins or miRNAs within BDEVs. For example, a single dose of ibuprofen in healthy volunteers led to acute changes in miRNA expression levels within neuron- and astrocyte-derived EVs (NDEVs and ADEVs, respectively) (Burrows et al., 2022, 2023). Similarly, an 8-week treatment with the SSRI escitalopram in patients with major depressive disorder (MDD) was associated with altered miRNA expression levels in NDEVs (Saeedi et al., 2021). Furthermore, a randomized clinical trial of the glucagon-like peptide-1 agonist exenatide in Parkinson's disease (PD) patients revealed changes in the expression and phosphorylation levels of several proteins in NDEVs during 48 weeks of treatment (Athauda et al., 2019). In addition to changes in BDEV cargo, some studies suggest that drug exposure may affect the biogenetic processes, as shown by altered NDEV sizes after chronic drug administration (Kumar et al., 2021; Saeedi et al., 2021). Collectively, these studies support the idea that BDEVs are sensitive to pharmacological manipulation and may serve as valuable sources of mechanistic biomarkers.

Second, some studies provide preliminary evidence that the effects of pharmacological treatments on BDEV physicochemical properties may be both dose- and time-dependent. For instance, the impact of ibuprofen on specific miRNA expression levels within NDEVs and ADEVs was greater at a 600 mg dose compared to 200 mg (Burrows et al., 2023). In the Exenatide trial for Parkinson's disease (PD), changes in protein expression and phosphorylation levels within NDEVs exhibited distinct time courses, with certain proteins showing altered expression 24 weeks into treatment, while others changed only at later timepoints (Athauda et al., 2019). Additionally, some of these alterations persisted 12 weeks after drug withdrawal, suggesting long-term shifts in molecular mechanisms underlying drug effects.

These observations underscore the dose- and time-sensitive nature of BDEV responses to drug exposure, supporting the potential of BDEVs for longitudinal assessments. It is also worth noting that time-dependent effects on BDEV cargo may not follow a linear trajectory; for example, a previous study in rodents indicated that EV miRNA levels could follow biphasic dynamics, initially increasing and then decreasing post-exposure (Motawi et al., 2018). Further studies are necessary to explore these complex relationships.

Third, these studies strongly suggest a correlation between drug-induced changes in BDEV physicochemical properties and functional changes in clinical outcomes, brain activity, or neurotoxicity. For instance, acute changes in specific miRNA expression levels within NDEVs and ADEVs following ibuprofen administration in healthy volunteers correlated with alterations in reward-related behavior and fMRI activation in specific brain regions (Burrows et al., 2022). Likewise, in patients with major depressive disorder (MDD) undergoing chronic escitalopram treatment, changes in a particular cluster of miRNAs within NDEVs predicted treatment response at an 8-week follow-up (Saeedi et al., 2021). Interestingly, the study also found that NDEV size was associated with MDD diagnosis and changed in relation to antidepressant treatment response.

In addition to therapeutic effects, alterations in BDEV size and content have been linked to neurotoxic effects of drugs. For example, long-term oxycodone self-administration in cynomolgus monkeys was associated with structural damage in the brain, as well as increased total EV size and altered levels of neurofilament light chain (NFL), amyloid-beta (Aβ1-42), and alpha-synuclein within NDEVs and ADEVs (Kumar et al., 2021). These observations suggest that BDEVs may offer the possibility to measure drug toxicity at the cellular and molecular level.

Fourth, changes in BDEV molecular cargo after drug exposure appear to align with the drug's presumed mechanism of action (MoA). For example, in human Exenatide trials, alterations in expression and phosphorylation levels of proteins associated with GLP-1 and insulin signaling pathways—such as IRS-1, Akt, and MAPK—were observed (Athauda et al., 2019). Similarly, miRNAs that showed differential expression following acute ibuprofen administration were selected based on their role in mediating the drug's anti-inflammatory effects within the CNS. In the case of antidepressant treatment (ADT), target prediction analysis of miRNAs predictive of treatment response highlighted biological pathways, including MAPK, that have been linked to antidepressant efficacy in preclinical depression models (Duman et al., 2007; Saeedi et al., 2021).

In summary, these studies reveal a clear link between the pharmacokinetic and pharmacodynamic (PK/PD) properties of drugs and changes in BDEV size and content. Additionally, the dose- and time-dependent dynamics of BDEV cargo changes align with functional and clinical outcomes. Thus, BDEVs offer a valuable platform for assessing the pharmacodynamic effects, signal transduction pathways, treatment responses, and neurotoxic properties of CNS drugs in an accessible, longitudinal manner. These findings support the use of BDEV cargo to investigate the neurobiological foundations of behavioral and cognitive effects, facilitating the integration of each UoA in RDoC-based human drug research.

## 4 Future challenges and opportunities for the use of in BDEV as mechanistic biomarkers in RDoC-based CNS drug discovery

Despite its potential, the use of BDEV cargo as mechanistic biomarkers in CNS drug discovery remains a relatively unexplored field, as evidenced by the limited number of studies. Consequently, it is difficult to draw firm conclusions, and numerous open questions need to be addressed to support broader applications. This section highlights these key challenges and outlines strategies to address them.

### 4.1 BDEV as platform to measure pharmacodynamic response

As these studies suggest, BDEVs could serve as a valuable tool for investigating pharmacodynamics in vivo, such as assessing drug-target interactions and cellular effects. Changes in the molecular content of BDEVs may, for example, potentially reflect the predicted mechanism of action (MoA) of a drug. However, this application is still in its early stages, and the exact pharmacodynamic mechanisms being studied within BDEVs remain unclear. In this regard, Pan et al. made significant progress with the development of the ExoSCOPE platform, which enables real-time monitoring of drug-target interactions—specifically, the EGFR inhibitor afatinib—in cancer patients using blood samples (Pan et al., 2021). These findings open up new possibilities for applying EVs in pharmacodynamic studies in humans.

For instance, BDEVs could be used to differentiate the pharmacodynamic profiles of drugs within the same class by tracking molecular changes in off-target signal transduction pathways. In such cases, increasing the drug dose might reveal stronger off-target effects within BDEV cargo. Similarly, BDEV cargo analysis could help investigate functional selectivity (i.e., biased agonism) of certain drugs at specific GPCR receptors. This could be achieved similarly to in vitro studies by measuring molecular changes in two distinct signal transduction pathways that are expected to respond differently to the drug (e.g., Gα versus β-arrestin pathways) (Rankovic et al., 2016; Thompson et al., 2015). Further studies are needed to expand the applications of BDEVs as pharmacodynamic biomarkers in CNS drug discovery.

### 4.2 Correlation between peripheral and central BDEV

Another important area is the need for additional preclinical studies to better establish the relationship between peripheral and central BDEVs, as well as their tissue of origin. Due to practical limitations, researchers have often relied on postmortem brain tissues from matched controls to test whether changes in peripheral BDEV cargo reflect those in the human brain. For example, in a study by Cheng et al. (2020) involving Alzheimer's disease (AD) patients, low correlations were observed between miRNA expression levels in EVs isolated from blood and those from postmortem brain tissues of matched controls. Notably, miRNAs that were differentially expressed between AD patients and healthy controls frequently showed inverse correlations between brain and blood samples. However, the study identified a set of miRNAs that were downregulated in both brain and blood of AD patients, suggesting that specific miRNAs in blood EVs could serve as reliable “liquid biopsy” markers for brain pathology. As the authors emphasized, these findings underscore the potential value of enriching BDEVs from blood—such as by immunocapturing cell-specific membrane markers—to enhance signal specificity. Overall, this study provides an initial indication of the potential for peripheral BDEVs to reflect central processes, highlighting the need for further research to validate these relationships.

### 4.3 Assessing the cellular origin of BDEV

Currently, it is not possible to determine the exact origin of BDEVs isolated from peripheral fluids, limiting the ability to link molecular changes in circulating BDEVs to specific brain regions (unless a BDEV subtype with unique brain origin is selected). Consequently, exploring biogenetic or physicochemical characteristics specific to EVs from certain brain regions may be valuable.

In a recent study, Huang et al. (2023) examined differences in EV density, size, morphology, and protein and RNA content across main brain regions, including the cerebellum, hippocampus, thalamus, and various neocortical lobes. They found that EVs isolated from the hippocampus and cerebellum showed higher expression of the tetraspanins CD9 and CD81 compared to other regions, as well as region-specific expression of neuron, astrocyte, and microglia markers. These findings suggest that certain brain regions may be over- or under-represented in cell-specific BDEV analyses from peripheral sources, depending on the surface markers chosen for immunoaffinity isolation. Additionally, the authors identified brain region-specific clusters of siRNA and miRNA expression through principal component analysis and unsupervised clustering. For instance, miR-137-3p and miR-744-5p were highly enriched exclusively in the orbitofrontal cortex, suggesting these as potential miRNA markers for EVs from that region. Since total EVs in this study were isolated from the brain tissue of a single individual using ultracentrifugation, further research in larger cohorts is needed to validate these findings. This would improve the application of peripheral BDEVs as biomarkers for monitoring drug effects in specific brain regions.

### 4.4 Blood-brain barrier crossing by BDEVs

A key consideration in using BDEVs as liquid biopsies for the brain is their ability to cross the blood-brain barrier (BBB). Although it is generally assumed that BDEVs can cross the BBB via transcytosis (Matsumoto et al., 2017), a recent review by Ramos-Zaldívar et al. (2022) highlights a lack of strong evidence to support this claim. Many studies to date have relied on in vitro or in vivo models that do not replicate the complex human microvasculature (Morad et al., 2019). For instance, García-Romero et al. (2017) demonstrated EV crossing of the BBB by identifying human-specific DNA sequences in peripheral EVs from a xenotransplant mouse model of human glioma-cancer stem cells. Similarly, Morad et al. (2019) used both mice injected with tumor EVs from breast cancer cells and in vitro static BBB models to suggest that EVs cross the BBB via transcytosis, facilitating brain metastasis.

While these studies indicate that BDEVs may cross the BBB, further research is required to clarify this mechanism using advanced in vitro and in vivo models, such as EV labeling in organoids and mammalian systems (Ramos-Zaldívar et al., 2022). For instance, researchers could examine BDEV cargo from the extracellular matrix of brain tissue samples using validated protocols (Cheng et al., 2020; Pérez-González et al., 2017; Vella et al., 2017) and correlate findings with BDEVs from peripheral fluids. Another approach could involve genetically engineering CNS cells to express fluorescent or chemiluminescent markers on EV membranes, allowing for direct tracking of BDEVs as they cross into the periphery (He et al., 2023). Such labeling studies would also enable researchers to quantify the proportion of BDEVs that cross the BBB—a measurement that remains undetermined. Importantly, because multiple biogenetic mechanisms exist, only a subset of EVs would be tagged with this approach.

It is also unclear whether BBB crossing dynamics differ between BDEV subtypes or are influenced by conditions associated with BBB disruption, such as Alzheimer's disease (Knox et al., 2022). Moreover, EV biogenesis and release rates can vary among individuals, influenced by factors such as fasting status, age, disease states, and environmental conditions. These individual differences in BDEV content should be taken into account when interpreting parental cell status in biomarker studies (van Heugten et al., 2022). Continued research is needed to better understand the factors influencing BDEV biogenesis and BBB crossing dynamics across different populations and conditions.

### 4.5 Selecting the molecular readout

There is currently no consensus on which type of EV cargo is best suited as a mechanistic biomarker. Ideally, the optimal approach would involve measuring multiple molecules within BDEVs using multi-omics techniques, such as a combination of LC-MS/MS and NGS to capture the whole proteome, transcriptome, and lipidome. Multi-omics datasets could then be integrated using Bayesian or network models (Subramanian et al., 2020) to provide an unbiased and comprehensive view of the molecular mechanisms underlying drug efficacy and safety. However, the heterogeneity and sheer size of multi-omics datasets, along with the lack of standardized methods for integrating them with clinical metadata, present significant challenges (Canzler et al., 2020; Subramanian et al., 2020). Moreover, multi-omics studies are often associated with high costs and complex data analysis, making it more cost-effective to focus on a fixed set of molecular readouts derived from these studies to measure drug effects in the CNS using BDEVs.

The selection of molecular readouts within BDEVs and the measurement methods used depend largely on the specific research question. For instance, proof-of-mechanism (PoM) studies with BDEVs have employed molecular readouts based on prior hypotheses regarding the drug's mechanism of action (MoA). These studies selected panels of either proteins or miRNAs to assess whether the drug engaged specific intracellular signaling pathways hypothesized to drive therapeutic effects (Athauda et al., 2019; Burrows et al., 2022, 2023), showing correlations between molecular expression changes and clinical outcomes. Alternatively, other studies have used omics approaches for the unbiased discovery of novel proteins or miRNAs associated with specific functional or clinical outcomes. For example, backward regression analysis of miRNA transcriptome data from NDEVs, measured via NGS, identified novel miRNA clusters associated with response to the SSRI citalopram in patients with major depressive disorder (MDD) (Saeedi et al., 2021). Bioinformatic analyses of these miRNAs yielded new insights into biological pathways that may mediate treatment response. Similarly, proteomic analysis of BDEVs revealed novel mechanisms underlying the neurotoxic effects of oxycodone self-administration in cynomolgus monkeys (Kumar et al., 2021).

In these studies, miRNA expression levels seem to be preferred as molecular readouts in BDEV research. Several factors may support this choice. First, EVs are highly enriched in miRNAs, with most serum miRNAs residing within exosomes (Gallo et al., 2012). Second, miRNAs are major regulators of gene expression, and changes in circulating miRNA levels have been linked to pathological conditions and environmental exposures, including drug administration (Krauskopf et al., 2015a, Krauskopf et al., 2015b; van den Berg et al., 2020; Wen et al., 2022). Importantly, the latter phenomenon was observed also in patients undergoing treatment with psychoactive medication, including antidepressants and antipsychotics (Pisanu et al., 2022; Martinez and Peplow, 2024). Third, miRNAs, particularly those within exosomes, are relatively stable and resistant to degradation by RNases and harsh conditions, making them suitable for long-term storage and transport compared to other RNA species or proteins (Ge et al., 2014; Sanz-Rubio et al., 2018). Finally, many miRNAs are evolutionarily conserved across species, enhancing their potential as translational biomarkers (Krauskopf et al., 2015a, Krauskopf et al., 2015b).

Despite these advantages, there are notable limitations when using miRNAs as molecular readouts in BDEV biomarker studies. First, miRNA expression levels exhibit high inter-individual variability, often necessitating large sample sizes to identify reliable biomarkers. Second, there is no consensus on methods for measuring differential miRNA expression from sequencing data or on how to interpret their functional roles. A single miRNA can target multiple mRNAs, affecting numerous genes (Riffo-Campos et al., 2016), and different studies use various prediction algorithms, making it challenging to draw conclusions about the biological pathways involved in drug effects without standardized analytic methods.

To address these challenges, initiatives for procedural harmonization, such as those proposed by the ISEV community for EV isolation and characterization, are needed for miRNA research. For example, the omics data analysis framework for regulatory application (R-ODAF) provides a user-friendly pipeline to improve reproducibility in analyzing raw transcriptomic data from microarrays and NGS (Verheijen et al., 2022). Additionally, researchers are encouraged to use curated miRNA-target interaction databases, such as miRTarBase, to interpret differentially expressed miRNAs (Hsu et al., 2010). These databases allow the selection of experimentally validated miRNA gene targets, identified through reliable methods like reporter assays, Western blotting, or qPCR. With validated gene targets, conventional pathway enrichment analysis using KEGG or GO terms can be conducted, facilitating robust functional insights.

## 5 Conclusion

**Fig. 3:**
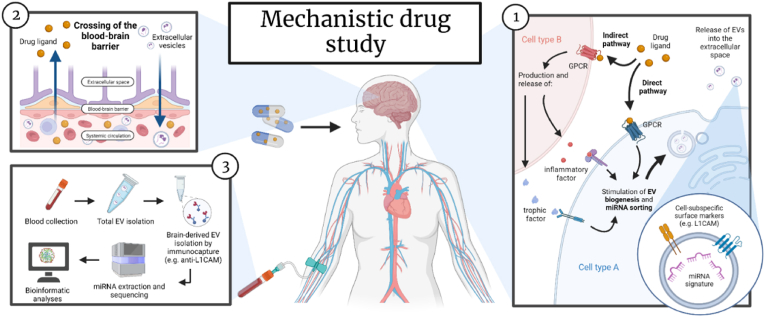
Conceptual overview of the use of miRNAs inside brain derived extracellular vesicles (BDVEs) as mechanistic biomarker in human drug studies. After administration, the drug molecule would penetrate inside the central nervous system (CNS) to bind with its target and trigger a biological response (1). This response can lead to the biogenesis of extracellular vesicles (EVs) and the sorting of specific pools of microRNAs (miRNAs) therein, via both direct and indirect mechanisms. Subsequently, miRNA-loaded EVs are released into the extracellular space and have the ability to cross the blood-brain barrier (BBB) and enter the peripheral circulation (2). The latter mechanisms, together with the presence of cell subspecific markers on their outer membrane, allows for the non-invasive collection and isolation of BDEVs from blood using immunoaffinity methods (3). The miRNA cargo of these BDEVs can then be extracted and sequenced using microarrays or next-generation sequencing tools. Finally, bioinformatic approaches such as pathway enrichment analysis can be employed to investigate the biological pathways activated by the drug. Created using BioRender.com.

RDoC-based drug development stands to benefit significantly from the advancement of non-invasive biomarkers capable of capturing molecular brain processes in living humans. This would enable clearer associations between each Unit of Analysis (UoA) within the RDoC matrix to obtain a comprehensive understanding of human behavior and its modulation by drugs. The literature reviewed here highlights the potential of analyzing circulating BDEV cargo, in particular miRNA content, to shed light on the effects of pharmacological treatments in specific cell types and their connections to functional or clinical outcomes. A conceptual overview of this approach is presented in Fig. 3.

Despite this promise, key challenges remain. Standardization of BDEV isolation, characterization, and validation is critical to their broader application as a source of mechanistic biomarkers. Therefore, adhering to standardized protocols, such as those proposed by the International Society for Extracellular Vesicles (Théry et al., 2018), will be crucial in improving reproducibility and expediting clinical translation. Furthermore, there is no consensus on the optimal molecular readouts for human BDEV studies. Although miRNAs have been widely used, their utility in elucidating biological mechanisms is constrained by technical and conceptual limitations. Future miRNA biomarker studies should be improved by implementing gold-standard practices in both experimental and data analysis methods (Valihrach et al., 2020). This includes rigorous validation protocols and standardized workflows aimed at enhancing consistency and reliability in miRNA research. The use of alternative and complementary approaches to miRNAs, such as integrating proteomics or other molecular measures, will therefore be crucial to enhance the depth of insights.

Considering these limitations, circulating BDEVs still represent an innovative biomarker strategy with the potential to transform RDoC-based drug discovery and development. By bridging molecular targets and functional clinical outcomes, BDEVs enable robust hypothesis testing across UoAs within PoM and PoC studies—capabilities that have been previously unattainable. Additionally, the use of BDEVs to investigate brain-related molecular processes would reduce reliance on postmortem tissue and animal models, aligning with the principles of the 3Rs (reduce, refine, replace) (Sneddon et al., 2017). Given their transformative potential, further research into the use of BDEV cargo as mechanistic biomarkers for the brain is strongly encouraged. This work could pave the way for a new era of precision in RDoC-based drug development, ultimately leading to novel and effective treatments for brain disorders.

## Contributions

IM led the research, conducted the primary literature review, and wrote the manuscript. RS supervised the project, providing guidance on research design and focus. JB, JR, JK, LdN, and YY reviewed the manuscript and contributed with valuable insights and comments to improve the work according to their respective area of expertise. The first author also would like to thank Dr. Houman Kahroba for the inspiring conversations that led to innovative insights on the matter.

## Declaration of generative AI and AI-assisted technologies in the writing process

Statement: during the preparation of the final (and subsequently accepted) version of the manuscript the authors used GPT-4o (OpenAI) in order to improve fluency and readability. After using this tool, the authors reviewed and edited the content as needed and take full responsibility for the content of the publication.

## Declaration of competing interest

There is no conflict of interest for our manuscript *“Brain-derived extracellular vesicles as non-invasive biomarkers in transdiagnostic CNS drug development“.*

## References

[bib1] Abels E.R., Breakefield X.O. (2016). Introduction to extracellular vesicles: biogenesis, RNA cargo selection, content, release, and uptake. Cell. Mol. Neurobiol..

[bib2] Abu-Asab M.S., Chaouchi M., Alesci S., Galli S., Laassri M., Cheema A.K., Atouf F., VanMeter J., Amri H. (2011). Biomarkers in the age of omics: time for a systems biology approach. OMICS A J. Integr. Biol..

[bib3] Akers J.C., Gonda D., Kim R., Carter B.S., Chen C.C. (2013). Biogenesis of extracellular vesicles (EV): exosomes, microvesicles, retrovirus-like vesicles, and apoptotic bodies. J. Neuro Oncol..

[bib4] Andreu Z., Yáñez-Mó M. (2014). Tetraspanins in extracellular vesicle formation and function. Front. Immunol..

[bib5] Angiolini F., Belloni E., Giordano M., Campioni M., Forneris F., Paronetto M.P., Lupia M., Brandas C., Pradella D., Di Matteo A., Giampietro C., Jodice G., Luise C., Bertalot G., Freddi S., Malinverno M., Irimia M., Moulton J.D., Summerton J., Ghigna C. (2019). A novel L1CAM isoform with angiogenic activity generated by NOVA2-mediated alternative splicing. Elife.

[bib6] Antoniou A., Auderset L., Kaurani L., Sebastian E., Zeng Y., Allahham M., Cases-Cunillera S., Schoch S., Gruendemann J., Fischer A., Schneider A. (2023). Neuronal extracellular vesicles and associated microRNAs induce circuit connectivity downstream BDNF. Cell Rep..

[bib7] Araldi E., Krämer-Albers E.-M., Hoen E.N.-t., Peinado H., Psonka-Antonczyk K.M., Rao P., van Niel G., Yáñez-Mó M., Nazarenko I. (2012). International society for extracellular vesicles: first annual meeting, april 17–21, 2012: ISEV-2012. J. Extracell. Vesicles.

[bib8] Aronson J.K., Ferner R.E. (2017). Biomarkers—a general review. Curr. Protoc. Pharmacol..

[bib9] Arvanitakis Z., Wang H.-Y., Capuano A.W., Khan A., Taïb B., Anokye-Danso F., Schneider J.A., Bennett D.A., Ahima R.S., Arnold S.E. (2020). Brain insulin signaling, alzheimer disease pathology, and cognitive function. Ann. Neurol..

[bib10] Athauda D., Gulyani S., Karnati H.k., Li Y., Tweedie D., Mustapic M., Chawla S., Chowdhury K., Skene S.S., Greig N.H., Kapogiannis D., Foltynie T. (2019). Utility of neuronal-derived exosomes to examine molecular mechanisms that affect motor function in patients with Parkinson disease: a secondary analysis of the exenatide-PD trial. JAMA Neurol..

[bib11] Badhwar A., Haqqani A.S. (2020). Biomarker potential of brain-secreted extracellular vesicles in blood in Alzheimer's disease. Alzheimer's Dementia: Diagnosis, Assessment & Disease Monitoring.

[bib12] Bağcı C., Sever-Bahcekapili M., Belder N., Bennett A.P.S., Erdener Ş E., Dalkara T. (2022). Overview of extracellular vesicle characterization techniques and introduction to combined reflectance and fluorescence confocal microscopy to distinguish extracellular vesicle subpopulations. Neurophotonics.

[bib13] Baldwin D.S., Kosky N. (2007). Off-label prescribing in psychiatric practice. Adv. Psychiatr. Treat..

[bib14] Basso M., Bonetto V. (2016). Extracellular vesicles and a novel form of communication in the brain. Front. Neurosci..

[bib15] Benedikter B.J., Bouwman F.G., Vajen T., Heinzmann A.C.A., Grauls G., Mariman E.C., Wouters E.F.M., Savelkoul P.H., Lopez-Iglesias C., Koenen R.R., Rohde G.G.U., Stassen F.R.M. (2017). Ultrafiltration combined with size exclusion chromatography efficiently isolates extracellular vesicles from cell culture media for compositional and functional studies. Sci. Rep..

[bib16] Borsboom D., Cramer A.O., Schmittmann V.D., Epskamp S., Waldorp L.J. (2011). The small world of psychopathology. PLoS One.

[bib17] Burrows K., Figueroa-Hall L.K., Kuplicki R., Stewart J.L., Alarbi A.M., Ramesh R., Savitz J.B., Teague T.K., Risbrough V.B., Paulus M.P. (2022). Neuronally-enriched exosomal microRNA-27b mediates acute effects of ibuprofen on reward-related brain activity in healthy adults: a randomized, placebo-controlled, double-blind trial. Sci. Rep..

[bib18] Burrows K., Figueroa-Hall L.K., Alarbi A.M., Stewart J.L., Kuplicki R., Tan C., Hannafon B.N., Ramesh R., Savitz J., Khalsa S., Teague T.K., Risbrough V.B., Paulus M.P. (2023). Association between inflammation, reward processing, and ibuprofen-induced increases of miR-23b in astrocyte-enriched extracellular vesicles: a randomized, placebo-controlled, double-blind, exploratory trial in healthy individuals. Brain, Behavior, & Immunity - Health.

[bib19] Cai Z., Li S., Matuskey D., Nabulsi N., Huang Y. (2019). PET imaging of synaptic density: a new tool for investigation of neuropsychiatric diseases. Neurosci. Lett..

[bib20] Cano A., Ettcheto M., Bernuz M., Puerta R., Esteban de Antonio E., Sánchez-López E., Souto E.B., Camins A., Martí M., Pividori M.I., Boada M., Ruiz A. (2023). Extracellular vesicles, the emerging mirrors of brain physiopathology. Int. J. Biol. Sci..

[bib21] Canzler S., Schor J., Busch W., Schubert K., Rolle-Kampczyk U.E., Seitz H., Kamp H., von Bergen M., Buesen R., Hackermüller J. (2020). Prospects and challenges of multi-omics data integration in toxicology. Arch. Toxicol..

[bib22] Cheng L., Vella L.J., Barnham K.J., McLean C., Masters C.L., Hill A.F. (2020). Small RNA fingerprinting of Alzheimer's disease frontal cortex extracellular vesicles and their comparison with peripheral extracellular vesicles. J. Extracell. Vesicles.

[bib24] Cohn W., Melnik M., Huang C., Teter B., Chandra S., Zhu C., McIntire L.B., John V., Gylys K.H., Bilousova T. (2021). Multi-omics analysis of microglial extracellular vesicles from human Alzheimer's disease brain tissue reveals disease-associated signatures. Front. Pharmacol..

[bib25] Cuthbert B.N. (2014). The RDoC framework: facilitating transition from ICD/DSM to dimensional approaches that integrate neuroscience and psychopathology. World Psychiatr..

[bib26] de Lange E.C.M., van den Brink W., Yamamoto Y., de Witte W.E.A., Wong Y.C. (2017). Novel CNS drug discovery and development approach: model-based integration to predict neuro-pharmacokinetics and pharmacodynamics. Expet Opin. Drug Discov..

[bib27] Deisseroth K. (2015). Optogenetics: 10 years of microbial opsins in neuroscience. Nat. Neurosci..

[bib28] (2013). Diagnostic and Statistical Manual of Mental Disorders : DSM-5.

[bib29] Dong L., Zieren R.C., Horie K., Kim C.-J., Mallick E., Jing Y., Feng M., Kuczler M.D., Green J., Amend S.R., Witwer K.W., de Reijke T.M., Cho Y.-K., Pienta K.J., Xue W. (2020). Comprehensive evaluation of methods for small extracellular vesicles separation from human plasma, urine and cell culture medium. J. Extracell. Vesicles.

[bib30] Duman C.H., Schlesinger L., Kodama M., Russell D.S., Duman R.S. (2007). A role for MAP kinase signaling in behavioral models of depression and antidepressant treatment. Biol. Psychiatr..

[bib31] Dutta S., Hornung S., Kruayatidee A., Maina K.N., Del Rosario I., Paul K.C., Wong D.Y., Duarte Folle A., Markovic D., Palma J.A., Serrano G.E., Adler C.H., Perlman S.L., Poon W.W., Kang U.J., Alcalay R.N., Sklerov M., Gylys K.H., Kaufmann H., Bitan G. (2021). α-Synuclein in blood exosomes immunoprecipitated using neuronal and oligodendroglial markers distinguishes Parkinson's disease from multiple system atrophy. Acta Neuropathol..

[bib32] Dutta S., Hornung S., Taha H.B., Bitan G. (2023). Biomarkers for parkinsonian disorders in CNS-originating EVs: promise and challenges. Acta Neuropathol..

[bib33] Egerton A. (2021). The potential of 1H-MRS in CNS drug development. Psychopharmacology.

[bib34] Fauré J., Lachenal G., Court M., Hirrlinger J., Chatellard-Causse C., Blot B., Grange J., Schoehn G., Goldberg Y., Boyer V., Kirchhoff F., Raposo G., Garin J., Sadoul R. (2006). Exosomes are released by cultured cortical neurones. Mol. Cell. Neurosci..

[bib35] Ferrer I., Martinez A., Boluda S., Parchi P., Barrachina M. (2008). Brain banks: benefits, limitations and cautions concerning the use of post-mortem brain tissue for molecular studies. Cell Tissue Bank..

[bib36] Fiandaca M.S., Kapogiannis D., Mapstone M., Boxer A., Eitan E., Schwartz J.B., Abner E.L., Petersen R.C., Federoff H.J., Miller B.L., Goetzl E.J. (2015). Identification of preclinical Alzheimer's disease by a profile of pathogenic proteins in neurally derived blood exosomes: a case-control study. Alzheimer's Dementia.

[bib38] Forgrave L.M., Ma M., Best J.R., DeMarco M.L. (2019). The diagnostic performance of neurofilament light chain in CSF and blood for Alzheimer's disease, frontotemporal dementia, and amyotrophic lateral sclerosis: a systematic review and meta-analysis. Alzheimer's Dementia: Diagnosis, Assessment & Disease Monitoring.

[bib39] Frank R., Hargreaves R. (2003). Clinical biomarkers in drug discovery and development. Nat. Rev. Drug Discov..

[bib40] Frühbeis C., Fröhlich D., Krämer-Albers E.M. (2012). Emerging roles of exosomes in neuron-glia communication. Front. Physiol..

[bib41] Frühbeis C., Fröhlich D., Kuo W.P., Amphornrat J., Thilemann S., Saab A.S., Kirchhoff F., Möbius W., Goebbels S., Nave K.A., Schneider A., Simons M., Klugmann M., Trotter J., Krämer-Albers E.M. (2013). Neurotransmitter-triggered transfer of exosomes mediates oligodendrocyte-neuron communication. PLoS Biol..

[bib42] Gallo A., Tandon M., Alevizos I., Illei G.G. (2012). The majority of MicroRNAs detectable in serum and saliva is concentrated in exosomes. PLoS One.

[bib43] García-Romero N., Carrión-Navarro J., Esteban-Rubio S., Lázaro-Ibáñez E., Peris-Celda M., Alonso M.M., Guzmán-De-Villoria J., Fernández-Carballal C., de Mendivil A.O., García-Duque S., Escobedo-Lucea C., Prat-Acín R., Belda-Iniesta C., Ayuso-Sacido A. (2017). DNA sequences within glioma-derived extracellular vesicles can cross the intact blood-brain barrier and be detected in peripheral blood of patients. Oncotarget.

[bib44] Ge Q., Zhou Y., Lu J., Bai Y., Xie X., Lu Z. (2014). miRNA in plasma exosome is stable under different storage conditions. Molecules.

[bib45] Gilleen J., Farah Y., Davison C., Kerins S., Valdearenas L., Uz T., Lahu G., Tsai M., Ogrinc F., Reichenberg A., Williams S.C., Mehta M.A., Shergill S.S. (2021). An experimental medicine study of the phosphodiesterase-4 inhibitor, roflumilast, on working memory-related brain activity and episodic memory in schizophrenia patients. Psychopharmacology.

[bib46] Goetzl E.J., Mustapic M., Kapogiannis D., Eitan E., Lobach I.V., Goetzl L., Schwartz J.B., Miller B.L. (2016). Cargo proteins of plasma astrocyte-derived exosomes in Alzheimer's disease. Faseb. J..

[bib47] Gomes D.E., Witwer K.W. (2022). L1CAM-associated extracellular vesicles: a systematic review of nomenclature, sources, separation, and characterization. Journal of Extracellular Biology.

[bib48] Griffiths P.D., Dobson B.R., Jones G.R., Clarke D.T. (1999). Iron in the basal ganglia in Parkinson's disease: an in vitro study using extended X-ray absorption fine structure and cryo-electron microscopy. Brain.

[bib49] Grimm O., Nägele M., Küpper-Tetzel L., de Greck M., Plichta M., Reif A. (2021). No effect of a dopaminergic modulation fMRI task by amisulpride and L-DOPA on reward anticipation in healthy volunteers. Psychopharmacology.

[bib50] Guedes V.A., Devoto C., Leete J., Sass D., Acott J.D., Mithani S., Gill J.M. (2020). Extracellular vesicle proteins and MicroRNAs as biomarkers for traumatic brain injury. Front. Neurol..

[bib51] Hack L.M., Williams L.M. (2021). Convergence Mental Health: A Transdisciplinary Approach to Innovation.

[bib52] Haspel J., Grumet M. (2003). The L1CAM extracellular region: a multi-domain protein with modular and cooperative binding modes. Front. Biosci..

[bib53] He Y., Xing Y., Jiang T., Wang J., Sang S., Rong H., Yu F. (2023). Fluorescence labeling of extracellular vesicles for diverse bio-applications in vitro and in vivo. Chem. Commun..

[bib54] Hessvik N.P., Llorente A. (2018). Current knowledge on exosome biogenesis and release. Cell. Mol. Life Sci..

[bib55] Hill A.F. (2019). Extracellular vesicles and neurodegenerative diseases. J. Neurosci..

[bib56] Hlavin M.L., Lemmon V. (1991). Molecular structure and functional testing of human L1CAM: an interspecies comparison. Genomics.

[bib57] Holcar M., Kandušer M., Lenassi M. (2021). Blood nanoparticles – influence on extracellular vesicle isolation and characterization. Front. Pharmacol..

[bib58] Holman N.S., Mosedale M., Wolf K.K., LeCluyse E.L., Watkins P.B. (2016). Subtoxic alterations in hepatocyte-derived exosomes: an early step in drug-induced liver injury?. Toxicol. Sci..

[bib59] Howes O.D., Mehta M.A. (2021). Challenges in CNS drug development and the role of imaging. Psychopharmacology (Berl).

[bib60] Hsu S.-D., Lin F.-M., Wu W.-Y., Liang C., Huang W.-C., Chan W.-L., Tsai W.-T., Chen G.-Z., Lee C.-J., Chiu C.-M., Chien C.-H., Wu M.-C., Huang C.-Y., Tsou A.-P., Huang H.-D. (2010). miRTarBase: a database curates experimentally validated microRNA–target interactions. Nucleic Acids Res..

[bib61] Huang C.-C., Yang P.-K., Huang Y.-S., Chen S.-U., Yang Y.-S., Chen M.-J. (2023). The role of circulating miRNAs in mechanism of action and prediction of therapeutic responses of metformin in polycystic ovarian syndrome. Fertil. Steril..

[bib62] ICD-11. (2018). World Health Organization.

[bib63] Im H., Shao H., Park Y.I., Peterson V.M., Castro C.M., Weissleder R., Lee H. (2014). Label-free detection and molecular profiling of exosomes with a nano-plasmonic sensor. Nat. Biotechnol..

[bib64] Insel T., Cuthbert B., Garvey M., Heinssen R., Pine D.S., Quinn K., Sanislow C., Wang P. (2010). Research domain criteria (RDoC): toward a new classification framework for research on mental disorders. Am. J. Psychiatr..

[bib65] Jannini T.B., Lorenzo G.D., Bianciardi E., Niolu C., Toscano M., Ciocca G., Jannini E.A., Siracusano A. (2022). Off-label uses of selective serotonin reuptake inhibitors (SSRIs). Curr. Neuropharmacol..

[bib66] Kang H., Kim J., Park J. (2017). Methods to isolate extracellular vesicles for diagnosis. Micro and Nano Systems Letters.

[bib67] Kantrowitz J.T., Milak M.S., Mao X., Shungu D.C., Mann J.J. (2016). D-cycloserine, an NMDA glutamate receptor Glycine site partial agonist, induces acute increases in brain glutamate plus glutamine and GABA comparable to ketamine. Am. J. Psychiatr..

[bib68] Kantrowitz J.T., Grinband J., Goff D.C., Lahti A.C., Marder S.R., Kegeles L.S., Girgis R.R., Sobeih T., Wall M.M., Choo T.-H., Green M.F., Yang Y.S., Lee J., Horga G., Krystal J.H., Potter W.Z., Javitt D.C., Lieberman J.A. (2020). Proof of mechanism and target engagement of glutamatergic drugs for the treatment of schizophrenia: RCTs of pomaglumetad and TS-134 on ketamine-induced psychotic symptoms and pharmacoBOLD in healthy volunteers. Neuropsychopharmacology.

[bib69] Knox E.G., Aburto M.R., Clarke G., Cryan J.F., O'Driscoll C.M. (2022). The blood-brain barrier in aging and neurodegeneration. Mol. Psychiatr..

[bib70] Kong L., He Q., Li Q., Schreiber R., Kaitin K.I., Shao L. (2023). Rapid progress in neuroimaging technologies fuels central nervous system translational medicine. Drug Discov. Today.

[bib71] Koníčková D., Menšíková K., Tučková L., Hényková E., Strnad M., Friedecký D., Stejskal D., Matěj R., Kaňovský P. (2022). Biomarkers of neurodegenerative diseases: biology, taxonomy, clinical relevance, and current research status. Biomedicines.

[bib72] Kraus V.B. (2018). Biomarkers as drug development tools: discovery, validation, qualification and use. Nat. Rev. Rheumatol..

[bib73] Krauskopf J., Caiment F., Claessen S.M., Johnson K.J., Warner R.L., Schomaker S.J., Burt D.A., Aubrecht J., Kleinjans J.C. (2015). Application of high-throughput sequencing to circulating microRNAs reveals novel biomarkers for drug-induced liver injury. Toxicol. Sci..

[bib74] Krauskopf J., Verheijen M., Kleinjans J.C., Kok T.M.d., Caiment F. (2015). Development and regulatory application of microRNA biomarkers. Biomarkers Med..

[bib75] Krystal A.D., Pizzagalli D.A., Smoski M., Mathew S.J., Nurnberger J., Lisanby S.H., Iosifescu D., Murrough J.W., Yang H., Weiner R.D., Calabrese J.R., Sanacora G., Hermes G., Keefe R.S.E., Song A., Goodman W., Szabo S.T., Whitton A.E., Gao K., Potter W.Z. (2020). A randomized proof-of-mechanism trial applying the 'fast-fail' approach to evaluating κ-opioid antagonism as a treatment for anhedonia. Nat. Med..

[bib76] Kumar A., Kim S., Su Y., Sharma M., Kumar P., Singh S., Lee J., Furdui C.M., Singh R., Hsu F.-C., Kim J., Whitlow C.T., Nader M.A., Deep G. (2021). Brain cell-derived exosomes in plasma serve as neurodegeneration biomarkers in male cynomolgus monkeys self-administrating oxycodone. EBioMedicine.

[bib77] Lai J.J., Chau Z.L., Chen S.Y., Hill J.J., Korpany K.V., Liang N.W., Lin L.H., Lin Y.H., Liu J.K., Liu Y.C., Lunde R., Shen W.T. (2022). Exosome processing and characterization approaches for research and technology development. Adv. Sci..

[bib78] Latifkar A., Hur Y.H., Sanchez J.C., Cerione R.A., Antonyak M.A. (2019). New insights into extracellular vesicle biogenesis and function. J. Cell Sci..

[bib79] Lewis A.J., Genoud C., Pont M., van de Berg W.D.J., Frank S., Stahlberg H., Shahmoradian S.H., Al-Amoudi A. (2019). Imaging of post-mortem human brain tissue using electron and X-ray microscopy. Curr. Opin. Struct. Biol..

[bib80] Li Y., Gui Y., Zhao M., Chen X., Li H., Tian C., Zhao H., Jiang C., Xu P., Zhang S., Ye S., Huang M. (2023). The roles of extracellular vesicles in major depressive disorder. Front. Psychiatr..

[bib81] Liangsupree T., Multia E., Riekkola M.-L. (2021). Modern isolation and separation techniques for extracellular vesicles. J. Chromatogr. A.

[bib82] Losurdo M., Grilli M. (2020). Extracellular vesicles, influential players of intercellular communication within adult neurogenic niches. Int. J. Mol. Sci..

[bib83] Luga V., Zhang L., Viloria-Petit A.M., Ogunjimi A.A., Inanlou M.R., Chiu E., Buchanan M., Hosein A.N., Basik M., Wrana J.L. (2012). Exosomes mediate stromal mobilization of autocrine Wnt-PCP signaling in breast cancer cell migration. Cell.

[bib84] Ma C., Jiang F., Ma Y., Wang J., Li H., Zhang J. (2019). Isolation and detection technologies of extracellular vesicles and application on cancer diagnostic. Dose Response.

[bib85] Margolis L., Sadovsky Y. (2019). The biology of extracellular vesicles: the known unknowns. PLoS Biol..

[bib86] Martinez B., Peplow P.V. (2024). MicroRNAs as potential biomarkers for diagnosis of schizophrenia and influence of antipsychotic treatment. Neural Regen Res.

[bib87] Martins T.S., Vaz M., Henriques A.G. (2023). A review on comparative studies addressing exosome isolation methods from body fluids. Anal. Bioanal. Chem..

[bib88] Matsumoto J., Stewart T., Banks W.A., Zhang J. (2017). The transport mechanism of extracellular vesicles at the blood-brain barrier. Curr. Pharmaceut. Des..

[bib89] Matthews H., Hanison J., Nirmalan N. (2016). “Omics”-Informed drug and biomarker discovery: opportunities, challenges and future perspectives. Proteomes.

[bib90] mCoumans F.A.W., Brisson A.R., Buzas E.I., Dignat-George F., Drees E.E.E., El-Andaloussi S., Emanueli C., Gasecka A., Hendrix A., Hill A.F., Lacroix R., Lee Y., van Leeuwen T.G., Mackman N., Mäger I., Nolan J.P., van der Pol E., Pegtel D.M., Sahoo S., Nieuwland R. (2017). Methodological guidelines to study extracellular vesicles. Circ. Res..

[bib91] Milà-Alomà M., Suárez-Calvet M., Molinuevo J.L. (2019). Latest advances in cerebrospinal fluid and blood biomarkers of Alzheimer's disease. Therapeutic Advances in Neurological Disorders.

[bib92] Morad G., Carman C.V., Hagedorn E.J., Perlin J.R., Zon L.I., Mustafaoglu N., Park T.E., Ingber D.E., Daisy C.C., Moses M.A. (2019). Tumor-derived extracellular vesicles breach the intact blood-brain barrier via transcytosis. ACS Nano.

[bib93] Morris S.E., Sanislow C.A., Pacheco J., Vaidyanathan U., Gordon J.A., Cuthbert B.N. (2022). Revisiting the seven pillars of RDoC. BMC Med..

[bib94] Motawi T.K., Mohamed M.R., Shahin N.N., Ali M.A.M., Azzam M.A. (2018). Time-course expression profile and diagnostic potential of a miRNA panel in exosomes and total serum in acute liver injury. Int. J. Biochem. Cell Biol..

[bib95] Mulcahy L.A., Pink R.C., Carter D.R.F. (2014). Routes and mechanisms of extracellular vesicle uptake. J. Extracell. Vesicles.

[bib96] Muraoka S., DeLeo A.M., Sethi M.K., Yukawa‐Takamatsu K., Yang Z., Ko J. (2020). Proteomic and biological profiling of extracellular vesicles from Alzheimer's disease human brain tissues. Alzheimer's Dementia.

[bib97] Mustapic M., Eitan E., Werner J.K., Berkowitz S.T., Lazaropoulos M.P., Tran J., Goetzl E.J., Kapogiannis D. (2017). Plasma extracellular vesicles enriched for neuronal origin: a potential window into brain pathologic processes. Front. Neurosci..

[bib98] Nagatsu T., Sawada M., Gerlach M., Deckert J., Double K., Koutsilieri E. (2007). Biochemistry of Postmortem Brains in Parkinson's Disease: Historical Overview and Future Prospects.

[bib99] Nelson N.C. (2015). A knockout experiment: disciplinary divides and experimental skill in animal behaviour genetics. Med. Hist..

[bib100] Niemantsverdriet E., Struyfs H., Duits F., Teunissen C.E., Engelborghs S., Deisenhammer F., Sellebjerg F., Teunissen C.E., Tumani H. (2015). Cerebrospinal Fluid in Clinical Neurology.

[bib101] Oraki Kohshour M., Papiol S., Delalle I., Rossner M.J., Schulze T.G. (2023). Extracellular vesicle approach to major psychiatric disorders. Eur. Arch. Psychiatr. Clin. Neurosci..

[bib102] Pan B.T., Johnstone R.M. (1983). Fate of the transferrin receptor during maturation of sheep reticulocytes in vitro: selective externalization of the receptor. Cell.

[bib103] Pan S., Zhang Y., Natalia A., Lim C.Z.J., Ho N.R.Y., Chowbay B., Loh T.P., Tam J.K.C., Shao H. (2021). Extracellular vesicle drug occupancy enables real-time monitoring of targeted cancer therapy. Nat. Nanotechnol..

[bib104] Pasanta D., He J.L., Ford T., Oeltzschner G., Lythgoe D.J., Puts N.A. (2023). Functional MRS studies of GABA and glutamate/Glx – a systematic review and meta-analysis. Neurosci. Biobehav. Rev..

[bib105] Pegtel D.M., Peferoen L., Amor S. (2014). Extracellular vesicles as modulators of cell-to-cell communication in the healthy and diseased brain. Phil. Trans. Biol. Sci..

[bib106] Pérez-González R., Gauthier S.A., Kumar A., Saito M., Saito M., Levy E., Hill A.F. (2017). Exosomes and Microvesicles: Methods and Protocols.

[bib107] Phillips M.L. (2012). Neuroimaging in psychiatry: bringing neuroscience into clinical practice. Br. J. Psychiatr..

[bib108] Pisanu C., Severino G., De Toma I., Dierssen M., Fusar-Poli P., Gennarelli M. (2022). Transcriptional biomarkers of response to pharmacological treatments in severe mental disorders: a systematic review. Eur. Neuropsychopharmacol.

[bib109] Pizzagalli D.A., Smoski M., Ang Y.S., Whitton A.E., Sanacora G., Mathew S.J., Nurnberger J., Lisanby S.H., Iosifescu D.V., Murrough J.W., Yang H., Weiner R.D., Calabrese J.R., Goodman W., Potter W.Z., Krystal A.D. (2020). Selective kappa-opioid antagonism ameliorates anhedonic behavior: evidence from the fast-fail trial in mood and anxiety Spectrum disorders (FAST-MAS). Neuropsychopharmacology.

[bib110] Pomatto M.A.C., Gai C., Bussolati B., Camussi G. (2017). Extracellular vesicles in renal pathophysiology. Front. Mol. Biosci..

[bib111] Potter W., Cuthbert B., Schreiber R. (2021). Modern CNS Drug Discovery : Reinventing the Treatment of Psychiatric and Neurological Disorders.

[bib112] Poulet G., Massias J., Taly V. (2019). Liquid biopsy: general concepts. Acta Cytol..

[bib113] Quek C., Hill A.F. (2017). The role of extracellular vesicles in neurodegenerative diseases. Biochem. Biophys. Res. Commun..

[bib115] Rajkowska G., Stockmeier C.A. (2013). Astrocyte pathology in major depressive disorder: insights from human postmortem brain tissue. Curr. Drug Targets.

[bib116] Ramos-Zaldívar H.M., Polakovicova I., Salas-Huenuleo E., Corvalán A.H., Kogan M.J., Yefi C.P., Andia M.E. (2022). Extracellular vesicles through the blood–brain barrier: a review. Fluids Barriers CNS.

[bib117] Rankovic Z., Brust T.F., Bohn L.M. (2016). Biased agonism: an emerging paradigm in GPCR drug discovery. Bioorg. Med. Chem. Lett.

[bib118] Raposo G., Stahl P.D. (2019). Extracellular vesicles: a new communication paradigm?. Nat. Rev. Mol. Cell Biol..

[bib119] Reddy P.H., Manczak M., Mao P., Calkins M.J., Reddy A.P., Shirendeb U. (2010). Amyloid-β and mitochondria in aging and Alzheimer's disease: implications for synaptic damage and cognitive decline. J. Alzheim. Dis..

[bib120] Riffo-Campos Á.L., Riquelme I., Brebi-Mieville P. (2016). Tools for sequence-based miRNA target prediction: what to choose?. Int. J. Mol. Sci..

[bib122] Roth B.L. (2016). DREADDs for neuroscientists. Neuron.

[bib123] Runz S., Keller S., Rupp C., Stoeck A., Issa Y., Koensgen D., Mustea A., Sehouli J., Kristiansen G., Altevogt P. (2007). Malignant ascites-derived exosomes of ovarian carcinoma patients contain CD24 and EpCAM. Gynecol. Oncol..

[bib124] Saeedi S., Nagy C., Ibrahim P., Théroux J.F., Wakid M., Fiori L.M., Yang J., Rotzinger S., Foster J.A., Mechawar N., Kennedy S.H., Turecki G. (2021). Neuron-derived extracellular vesicles enriched from plasma show altered size and miRNA cargo as a function of antidepressant drug response. Mol. Psychiatr..

[bib125] Saheera S., Jani V.P., Witwer K.W., Kutty S. (2021). Extracellular vesicle interplay in cardiovascular pathophysiology. Am. J. Physiol. Heart Circ. Physiol..

[bib126] Sakayori T., Tateno A., Arakawa R., Kim W.-c., Okubo Y. (2021). Evaluation of dopamine D3 receptor occupancy by blonanserin using [11C]-(+)-PHNO in schizophrenia patients. Psychopharmacology.

[bib127] Samatov T.R., Wicklein D., Tonevitsky A.G. (2016). L1CAM: cell adhesion and more. Prog. Histochem. Cytochem..

[bib128] Sanz-Rubio D., Martin-Burriel I., Gil A., Cubero P., Forner M., Khalyfa A., Marin J.M. (2018). Stability of circulating exosomal miRNAs in healthy subjects. Sci. Rep..

[bib129] Sedgwick A.E., D'Souza-Schorey C. (2018). The biology of extracellular microvesicles. Traffic.

[bib130] Sele M., Wernitznig S., Lipovšek S., Radulović S., Haybaeck J., Birkl-Toeglhofer A.M., Wodlej C., Kleinegger F., Sygulla S., Leoni M., Ropele S., Leitinger G. (2019). Optimization of ultrastructural preservation of human brain for transmission electron microscopy after long post-mortem intervals. Acta Neuropathologica Communications.

[bib131] Shao S., Fang H., Li Q., Wang G. (2020). Extracellular vesicles in inflammatory skin disorders: from pathophysiology to treatment. Theranostics.

[bib132] Shi M., Liu C., Cook T.J., Bullock K.M., Zhao Y., Ginghina C., Li Y., Aro P., Dator R., He C., Hipp M.J., Zabetian C.P., Peskind E.R., Hu S.-C., Quinn J.F., Galasko D.R., Banks W.A., Zhang J. (2014). Plasma exosomal α-synuclein is likely CNS-derived and increased in Parkinson's disease. Acta Neuropathol..

[bib133] Silverman J.L., Nithianantharajah J., Der-Avakian A., Young J.W., Sukoff Rizzo S.J. (2020). Lost in translation: at the crossroads of face validity and translational utility of behavioral assays in animal models for the development of therapeutics. Neurosci. Biobehav. Rev..

[bib134] Skog J., Würdinger T., van Rijn S., Meijer D.H., Gainche L., Curry W.T., Carter B.S., Krichevsky A.M., Breakefield X.O. (2008). Glioblastoma microvesicles transport RNA and proteins that promote tumour growth and provide diagnostic biomarkers. Nat. Cell Biol..

[bib135] Sneddon L.U., Halsey L.G., Bury N.R. (2017). Considering aspects of the 3Rs principles within experimental animal biology. J. Exp. Biol..

[bib136] Subramanian I., Verma S., Kumar S., Jere A., Anamika K. (2020). Multi-omics data integration, interpretation, and its application. Bioinf. Biol. Insights.

[bib137] Sung C.C., Chen M.H., Lin Y.C., Lin Y.C., Lin Y.J., Yang S.S., Lin S.H. (2021). Urinary extracellular vesicles for renal tubular transporters expression in patients with gitelman syndrome. Front. Med..

[bib138] Szatanek R., Baj-Krzyworzeka M., Zimoch J., Lekka M., Siedlar M., Baran J. (2017). The methods of choice for extracellular vesicles (EVs) characterization. Int. J. Mol. Sci..

[bib139] Théry C., Witwer K.W., Aikawa E., Alcaraz M.J., Anderson J.D., Andriantsitohaina R., Antoniou A., Arab T., Archer F., Atkin-Smith G.K., Ayre D.C., Bach J.M., Bachurski D., Baharvand H., Balaj L., Baldacchino S., Bauer N.N., Baxter A.A., Bebawy M., Zuba-Surma E.K. (2018). Minimal information for studies of extracellular vesicles 2018 (MISEV2018): a position statement of the International Society for Extracellular Vesicles and update of the MISEV2014 guidelines. J. Extracell. Vesicles.

[bib140] Thompson G.L., Lane J.R., Coudrat T., Sexton P.M., Christopoulos A., Canals M. (2015). Biased agonism of endogenous opioid peptides at the <em>μ</em>-Opioid receptor. Mol. Pharmacol..

[bib141] Thul P.J., Lindskog C. (2018). The human protein atlas: a spatial map of the human proteome. Protein Sci..

[bib142] Tian J., Casella G., Zhang Y., Rostami A., Li X. (2020). Potential roles of extracellular vesicles in the pathophysiology, diagnosis, and treatment of autoimmune diseases. Int. J. Biol. Sci..

[bib143] Tian C., Stewart T., Hong Z., Guo Z., Aro P., Soltys D., Pan C., Peskind E.R., Zabetian C.P., Shaw L.M., Galasko D., Quinn J.F., Shi M., Zhang J. (2022). Blood extracellular vesicles carrying synaptic function- and brain-related proteins as potential biomarkers for Alzheimer's disease. Alzheimers Dement.

[bib144] Tio P., Epskamp S., Noordhof A., Borsboom D. (2016). Mapping the manuals of madness: comparing the ICD-10 and DSM-IV-TR using a network approach. Int. J. Methods Psychiatr. Res..

[bib145] Tiwari S., Kumar V., Randhawa S., Verma S.K. (2021). Preparation and characterization of extracellular vesicles. Am. J. Reprod. Immunol..

[bib146] Tricklebank M.D., Robbins T.W., Simmons C., Wong E.H.F. (2021). Time to re-engage psychiatric drug discovery by strengthening confidence in preclinical psychopharmacology. Psychopharmacology.

[bib147] Tronel C., Largeau B., Santiago Ribeiro M.J., Guilloteau D., Dupont A.-C., Arlicot N. (2017). Molecular targets for PET imaging of activated microglia: the current situation and future expectations. Int. J. Mol. Sci..

[bib148] Valihrach L., Androvic P., Kubista M. (2020). Circulating miRNA analysis for cancer diagnostics and therapy. Mol. Aspect. Med..

[bib149] van den Berg M.M.J., Krauskopf J., Ramaekers J.G., Kleinjans J.C.S., Prickaerts J., Briedé J.J. (2020). Circulating microRNAs as potential biomarkers for psychiatric and neurodegenerative disorders. Prog. Neurobiol..

[bib150] van der Doef T.F., Domingo S.Z., Jacobs G.E., Drevets W.C., Marston H.M., Nathan P.J. (2018). New approaches in psychiatric drug development. Eur. Neuropsychopharmacol.

[bib151] Van Deun J., Mestdagh P., Agostinis P., Akay Ö., Anand S., Anckaert J., Martinez Z.A., Baetens T., Beghein E., Bertier L., Berx G., Boere J., Boukouris S., Bremer M., Buschmann D., Byrd J.B., Casert C., Cheng L., Cmoch A., Hendrix A. (2017). EV-TRACK: transparent reporting and centralizing knowledge in extracellular vesicle research. Nat. Methods.

[bib152] van Eijndhoven P., Collard R., Vrijsen J., Geurts D.E.M., Vasquez A.A., Schellekens A., van den Munckhof E., Brolsma S., Duyser F., Bergman A., van Oort J., Tendolkar I., Schene A. (2022). Measuring integrated novel dimensions in neurodevelopmental and stress-related mental disorders (MIND-SET): protocol for a cross-sectional comorbidity study from a research domain criteria perspective. JMIRx Med.

[bib153] van Heugten M.H., Hoorn E.J., Fenton R.A. (2022). Urinary extracellular vesicles: does cargo reflect tissue?. Curr. Opin. Nephrol. Hypertens..

[bib154] van Niel G., D'Angelo G., Raposo G. (2018). Shedding light on the cell biology of extracellular vesicles. Nat. Rev. Mol. Cell Biol..

[bib155] Vella L.J., Scicluna B.J., Cheng L., Bawden E.G., Masters C.L., Ang C.-S., Willamson N., McLean C., Barnham K.J., Hill A.F. (2017). A rigorous method to enrich for exosomes from brain tissue. J. Extracell. Vesicles.

[bib156] Verheijen M.C., Meier M.J., Asensio J.O., Gant T.W., Tong W., Yauk C.L., Caiment F. (2022). R-ODAF: omics data analysis framework for regulatory application. Regul. Toxicol. Pharmacol..

[bib157] Volpert O., Gershun E., Elgart K., Kalia V., Wu H., Baccarelli A., Eren E., Kapogiannis D., Verma A., Levin A., Eitan E. (2022). Novel modification of Luminex assay for characterization of extracellular vesicle populations in biofluids. bioRxiv.

[bib158] Waldvogel H.J., Curtis M.A., Baer K., Rees M.I., Faull R.L.M. (2006). Immunohistochemical staining of post-mortem adult human brain sections. Nat. Protoc..

[bib159] Wallensten J., Nager A., Åsberg M., Borg K., Beser A., Wilczek A., Mobarrez F. (2021). Leakage of astrocyte-derived extracellular vesicles in stress-induced exhaustion disorder: a cross-sectional study. Sci. Rep..

[bib160] Wang J., Guo R., Yang Y., Jacobs B., Chen S., Iwuchukwu I., Gaines K.J., Chen Y., Simman R., Lv G., Wu K., Bihl J.C. (2016). The novel methods for analysis of exosomes released from endothelial cells and endothelial progenitor cells. Stem Cell. Int..

[bib161] Welsh J.A., Goberdhan D.C., O'Driscoll L., Buzas E.I., Blenkiron C., Bussolati B. (2024). Minimal information for studies of extracellular vesicles (MISEV2023): from basic to advanced approaches. J. Extracell. Vesicles.

[bib162] Wen Q., Verheijen M., Wittens M.M.J., Czuryło J., Engelborghs S., Hauser D., van Herwijnen M.H.M., Lundh T., Bergdahl I.A., Kyrtopoulos S.A., de Kok T.M., Smeets H.J.M., Briedé J.J., Krauskopf J. (2022). Lead-exposure associated miRNAs in humans and Alzheimer's disease as potential biomarkers of the disease and disease processes. Sci. Rep..

[bib163] Willis C.M., Ménoret A., Jellison E.R., Nicaise A.M., Vella A.T., Crocker S.J. (2017). A refined bead-free method to identify astrocytic exosomes in primary glial cultures and blood plasma. Front. Neurosci..

[bib164] Wu Q., Poulsen S.B., Murali S.K., Grimm P.R., Su X.T., Delpire E., Welling P.A., Ellison D.H., Fenton R.A. (2021). Large-scale proteomic assessment of urinary extracellular vesicles highlights their reliability in reflecting protein changes in the kidney. J. Am. Soc. Nephrol..

[bib165] Xu R., Greening D.W., Zhu H.-J., Takahashi N., Simpson R.J. (2016). Extracellular vesicle isolation and characterization: toward clinical application. J. Clin. Investig..

[bib166] Yang F., Liao X., Tian Y., Li G. (2017). Exosome separation using microfluidic systems: size-based, immunoaffinity-based and dynamic methodologies. Biotechnol. J..

[bib167] You Y., Ikezu T. (2019). Emerging roles of extracellular vesicles in neurodegenerative disorders. Neurobiol. Dis..

[bib168] You Y., Muraoka S., Jedrychowski M.P., Hu J., McQuade A.K., Young‐Pearse T. (2022). Human neural cell type‐specific extracellular vesicle proteome defines disease‐related molecules associated with activated astrocytes in Alzheimer's disease brain. J. Extracell. Vesicles.

[bib169] You Y., Zhang Z., Sultana N., Ericsson M., Martens Y.A., Sun M., Kanekiyo T., Ikezu S., Shaffer S.A., Ikezu T. (2023). ATP1A3 as a target for isolating neuron-specific extracellular vesicles from human brain and biofluids. Sci. Adv..

[bib170] Yousif G., Qadri S., Parray A., Akhthar N., Shuaib A., Haik Y. (2022). Exosomes derived neuronal markers: immunoaffinity isolation and characterization. NeuroMolecular Med..

[bib171] Yu Z., Shi M., Stewart T., Fernagut P.O., Huang Y., Tian C., Dehay B., Atik A., Yang D., De Giorgi F., Ichas F., Canron M.H., Ceravolo R., Frosini D., Kim H.J., Feng T., Meissner W.G., Zhang J. (2020). Reduced oligodendrocyte exosome secretion in multiple system atrophy involves SNARE dysfunction. Brain.

[bib172] Zhang Y., Xu C. (2022). Effects of exosomes on adult hippocampal neurogenesis and neuropsychiatric disorders. Mol. Biol. Rep..

[bib173] Zhang Z., Yu K., You Y., Jiang P., Wu Z., DeTure M.A. (2023). Comprehensive characterization of human brain‐derived extracellular vesicles using multiple isolation methods: implications for diagnostic and therapeutic applications. J. Extracell. Vesicles.

