# Supplementary material for: Harnessing brain-derived extracellular vesicles to support RDoC-based drug development
Source: Neurosci Appl. 2024 Dec 15;4:105406. doi: 10.1016/j.nsa.2024.105406 (PMC12244223; doi:10.1016/j.nsa.2024.105406)
Supplement: Multimedia component 3 [file mmc3.docx]

# Supplement

## Appendix 1: Full description of reviewed studies

Burrows and colleagues (2022 & 2023) investigated the use of miRNAs inside neuron-derived EVs (NDEVs) and astrocyte-derived EVs (ADEVs) to test the hypothesis that the non-steroidal anti-inflammatory drug ibuprofen modulates reward-related processing in humans by altering the expression of genes involved in neuroinflammatory processes. The study included twenty healthy adult subjects which underwent treatment with placebo, 200 mg, or 600 mg of ibuprofen on three separate sessions according to a within-subject design. During test days, fMRI scans during a monetary incentive delay task and blood samples were taken 1- and 5-hours post drug administration, respectively. Total EVs were isolated form blood serum using the ExoQuick precipitation assay, followed by immunocapturing of neuron- and astrocytes-derived EVs using anti-L1CAM, and anti-ACSA-1 biotinylated antibodies coupled to streptavidin-coated magnetic beads. The resulting BDEV populations were characterized by NTA, flow cytometry, and Western Blot analysis. MiRNA purification was achieved using the Qiagen miRNeasy Micro Kit, and concentration of small RNA was assessed using the Agilent Small RNA kit. Finally, miRNAs were sequenced using next-generation sequencing (NGS) on the Illumina platform. Despite NGS providing full transcriptome datasets, the authors focused on a specific panel of miRNAs, in particular miR-27b-3p and miR-320b. In fact, these miRNAs exhibited higher expression in NDEVs and ADEVs after ibuprofen administration, an effect which was dose-dependent in most cases. Importantly, results of a moderator analysis showed main effects of miR-27b-3p expression levels in NDEVs on activation in specific brain areas during gain or loss anticipation phases in the MID task. Moreover, increased AEEV miR-23b-3p expression levels correlated with changes in circulating inflammatory markers, such as serum tumor-necrosis factor alpha (TNF-alpha) and interleukin-17A. Taken together, this study shows that miRNAs inside BDEV represent a valuable tool to prove the MoA of drug in the CNS at the level of inflammation, and its relationship to functional changes in brain activity and behavior.

Saeedi and colleagues (2021) investigated the use of miRNAs inside NDEVs to predict antidepressant treatment response (ADT) in patients with major depressive disorder (MDD). The study included 20 healthy control and 40 MDD patients that underwent an 8-week treatment with the selective serotonin reuptake inhibitor (SSRI) escitalopram. At the end of the treatment period, these patients were subdivided into responders and non-responders, as determined by >50% decrease in the Montgomery-Asberg Depression Rating Scale (MADRS) score. Blood plasma samples were taken at both baseline and at the end of treatment. From these samples, total EVs were isolated by SEC using the qEV 70 columns (Izon Science), followed by immunocapture of NDEVs using anti-L1CAM biotinylated antibodies coupled with Streptavidin-plus ultra-link resin (Thermo). The size and concentration of the resulting NDEV populations were characterized by Tunable Resistive Pulse Sensing (TRPS) using the qNano platform (Izon). Next, NDEVs were visualized using transmission electron microscopy. To measure the purity of their NDEV populations, the presence of exosomal and neuronal markers in total EVs, NDEVs, and protein fractions collected after EV SEC was checked using Western Blot. Moreover, an innovative approach was used to prove enrichment of neuronal miRNAs inside NDEVs. In fact, the authors showed a significant overlap between miRNAs inside NDEVs and human cortical brain tissue. Moreover, target prediction algorithms performed on consensus binding motifs of these miRNAs showed significant enrichment of target genes in the brain.

The authors observed reduced average sizes of both total and neuron-derived EV in MDD patients before the start of ADT, which changed over the course of treatment as a function of treatment response. This result was later confirmed in an independent replication cohort and in vitro cell lines, including human neural progenitor cells and HEK293T cells. More significantly, the authors observed a change over time in the expression level of nine miRNAs, three of which were predictive of response according to stepwise regression analysis, namely miR-21-5p, miR-30d-5p, and miR-486-5p. To validate these results, expression levels of these miRNAs were measured in the same NDEV samples using qPCR. Moreover, the results were confirmed in NDEVs from a separate cohort of MDD patients undergoing ADT with escitalopram, which replicated the observation for miR-30d-5p and miR-486-5p, yet not for miR-21-5p. Target prediction analysis of these miRNAs revealed gene targets that are highly enriched in brain tissue, and which may be responsible for mediating ADT, like the MAPK pathway and axon guidance. To validate these results, the authors looked at comparable changes in the expression levels of genes of the predicted targets in the anterior cingulate cortex (ACC) of people who died by suicide during MDD. Taken together, these results suggest that changes in miRNA expression inside NDEV can be predictive of long-term ADT response, while providing mechanistic insights in the molecular and biological mechanisms underlying this response. Moreover, this study highlights the advantage of using independent cohort samples, in vitro models, and brain tissues biopsies to confirm and validate the initial findings.

Athauda et al. (2019) explored the use of protein content of NDEVs to investigate the mechanism of action of the glucagon-like peptide 1 agonist Exenatide in Parkinson’s disease (PD). The study was a randomized controlled trial (RCT) including 60 PD patients which were randomized to self-inject either placebo (n=29) or 2 mg exenatide (n=31) once weekly for 48 weeks. Blood samples were collected at baseline, week 24, week 48 (end of treatment), and week 60 (one week after drug withdrawal). For NDEV isolation, the authors used the validated 2-step protocol of the Goetzl group previously mentioned, consisting of an initial precipitation of total EVs followed by immunoprecipitation of L1CAM+ EVs. Although the authors refer to Mustapic et al. (2017) for a detailed description of the methods, the publication does not provide information on the type of blood samples used (serum v. plasm) and any NDEV characterization data such as EV size and density, and the presence of exosomal and neuronal markers. With the resulting NDEV populations, the authors quantified an *a priori* selected panel of protein related to the presumed MoA of exenatide. This panel included proteins that are part of the GLP-1 and insulin signaling, such as various phosphorylated IRS-1 proteins and downstream effectors of the Akt and MAPK pathway. These proteins were quantified in lysed NDEVs by electrochemiluminescence using the Mesoscale discovery platform.

The results showed changes in the levels of various NDEVs protein in patients undergoing treatment with exenatide compared to placebo. Importantly, the magnitude and time-course of these changes differed between various proteins, some of which were not predicted by authors and provided novel insights on the MoA of exenatide. Moreover, multiple linear regression analysis showed that the changes in clinical outcome at the end of treatment and after cessation were determined by changes in the level of certain proteins within NDEVs, such as IRS-1 p-S616, t-mTOR, and p-mTOR S2448. Taken together, these results show that measuring changes in expression and phosphorylation level of proteins inside NDEVs may be used to assert the MoA of a drug inside the CNS and potentially to predict future treatment outcomes. Yet, we again emphasize the need to provide thorough description of NDEV isolation and characterization protocols to support method standardization and comparison between studies.

Kumar et al. (2021) used a panel of various cell specific BDEVs to thoroughly investigate the neurotoxic effects of long-term (3 years) oxycodone self-administration in six adult male Cynomolgus monkeys. In this study, structural neurodegeneration was measured by collecting structural brain imaging data using a 3 Tesla MRI on ketamine-sedated subject. Blood plasma samples were collected in K3-EDTA tubes, which were immediately centrifuged and stored at -80C. Total EV were isolated from these samples using the ExoQuick precipitation methods, followed by enrichment of neuron-, astrocyte-, and microglia-derived EVs by immunocapture using streptavidin-coated magnetic beads coupled to biotinylated antibodies against L1CAM, GLAST, and TMEM119, respectively. Resulting BDEVs populations were characterized using NTA, flow cytometry, and Western Blot. Within these BDEVs, expression levels of neurodegeneration miRNA and protein validated markers were measured using real-time PCR and ELISA, respectively. Additionally, proteomic analysis using liquid chromatography coupled to mass spectrometry (LC-MS) was for unbiased identification of novel protein markers for neurodegeneration inside BDEVs. Finally, the NF-kb activation assay was performed on THP-1 Lucia NF-kb monocyte cells to measure inflammatory response to each BDEV.

The results of this study show that long-term oxycodone self-administration was associated with an increase in the size of total EVs. Changes in the levels of NFL, Aβ1-42, and α-synuclein levels were observed in NDEVs and ADEVs of oxycodone-treated animals compared to vehicle. Yet, no differences in α-synuclein and NFL levels were observed in total EVs, thus supporting the notion that looking at changes of protein expression inside specific EV subpopulations compared to total EVs may increase the sensitivity of the assay. Moreover, some of these changes correlated with reductions in gray matter volumes in specific brain areas, such as the frontal and parietal lobes. Similarly, the authors observed changes in the expression levels of neurodegeneration-associated miRNAs inside BDEVs, such as miR-125-5p, miR-16-5p, and miR-339-3p. Importantly, these changes varied depending on the BDEV type.

Proteomic analysis revealed changes in the expression levels of several proteins in NDEV, MDEVs, and total EVs between control and oxycodone-treated animals. Pathway analyses on higher abundance proteins inside NDEVs and ADEVs revealed association with molecular processes that are known to play a role in addiction-related behaviors and neuroinflammation. Among these, involvement of the glucocorticoid receptor (GR) signaling pathway in the neuroinflammatory response to oxycodone was subsequently tested in vitro. Remarkably, the authors showed that exposure of astrocytes to NDEVs isolated from oxycodone-treated animals led to an increase in GR nuclear translocation compared to cells treated with control NDEVs. Similarly, the involvement of inflammatory pathways in the neurotoxic effects of oxycodone was confirmed by higher NF-kb activity in THP1 cells exposed to NDEVs, ADEVs, or MDEVs from oxycodone-treated animals compared to control.

This study shows that BDEVs can provide valuable insights into the neurotoxic effects of drugs acting in the CNS. Moreover, it highlights the advantage of using proteomic analysis, like LC-MS, on BDEVs to discover biological processes underlying these effects in an unbiased manner, i.e. without relying on *a priori* selected protein markers. Also, it provides novel approaches for the validation of these findings using in vitro models, similarly to Saeedi et al. (2021). Yet, it should also be mentioned that Western Blot characterization of exosomal markers in total EVs in this study showed apparent semi-quantitative difference in the signal between control and oxycodone conditions, which have not been highlighted by the authors. In fact, Western Blot images show higher expression of exosomal markers in the oxycodone total EVs, especially for the markers Alix, and CD9. Together with differences in size but similar concentrations, this result may suggest that total EV isolation resulted in different pools of EVs between the two conditions. Therefore, replication of these results in a different cohort of subjects would be needed to validate these findings. Moreover, since WB characterization with Western Blot was not performed on the resulting BDEV subpopulations, it is unclear whether this observation applies to the latter as well. Yet, this information would have been valuable to determine whether BDEV isolation from blood of control and oxycodone-treated animals resulted in EV subtypes with comparable purity, thus allowing for proper comparison of proteomic and sequencing data.

## Appendix 2: figure 2 BioRender publication license

## Appendix 3: figure 3 BioRender publication license
